# Engineering Tumor‐Specific Nanotheranostic Agent with MR Image‐Guided NIR‐II & ‐III Photodynamic Therapy to Combat Against Deeply Seated Orthotopic Glioblastoma

**DOI:** 10.1002/smsc.202400191

**Published:** 2024-07-14

**Authors:** Karthik Nuthalapati, Raviraj Vankayala, Munusamy Shanmugam, Suresh Thangudu, Chi‐Shiun Chiang, Kuo Chu Hwang

**Affiliations:** ^1^ Department of Chemistry and Institute of Analytical and Environmental Sciences National Tsing Hua University Hsinchu 30013 Taiwan; ^2^ Department of Bioscience and Bioengineering Interdisciplinary Research Platform Smart Healthcare Indian Institute of Technology Jodhpur Karwar 342030 India; ^3^ Department of Biomedical Engineering and Environmental Sciences National Tsing Hua University Hsinchu 30013 Taiwan

**Keywords:** deeply seated tumors, glioblastoma, magnetic resonance imaging, NIR photodynamic therapy, NIR‐II and ‐III biological windows, reactive oxygen species

## Abstract

Glioblastoma multiforme (GBM) is one of the most aggressive, incurable, and difficult‐to‐treat malignant brain tumor with very poor survival rates. The gold standard in treating GBMs includes neurosurgical resection of the tumor, followed by the chemotherapy and radiotherapy. However, these strategies remain ineffective in treating patients with GBMs, as tumor recurrence always occur in most cases. Therefore, it remains a grand challenge to develop an effective strategy to combat orthotopic glioblastoma with simultaneous imaging capabilities to monitor the therapeutic outcomes. To tackle this challenge, this study demonstrates, for the first time, that a tumor‐specific europium hexaboride (EuB_6_)‐based nanomedicine surface‐modified with RGD‐K peptide to target α_v_β_3_ integrin receptors overexpressed on the glioblastoma cells. Further, EuB_6_@RGD‐K NPs are able to exert theranostic capabilities to effectively diagnose and combat difficult‐to‐treat orthotopic glioblastoma tumors using NIR‐II 1064 nm and NIR‐III 1550 nm photodynamic therapy (NIR PDT) effects. In the in vivo experiments, the average half‐life of 55 d for mice treated with EuB_6_@RGD‐K NPs and exposed to NIR‐III 1550 nm light irradiation is far higher than that of EuB_6_@RGD‐K NPs exposed to NIR‐II 1064 nm light irradiation (25 d), PBS‐treated mice (20 d) and EuB_6_@RGD‐K NPs‐treated mice (no light irradiation, 18 d). To the best of our knowledge, this work represents the first example for destructing murine brain tumors via multi‐functional tumor‐specific europium hexaboride‐based nanotheranostic agent to mediate MR imaging‐guided NIR‐II/‐III photodynamic therapy.

## Graphical abstract

EuB_6_ NPs are surface‐modified with RGD‐K peptides and used to exert NIR‐II/‐III photodynamic therapy for treating deeply seated orthotopic glioblastoma under MR imaging‐guidance, leading to significantly prolonged average half‐life of 55 d for mice treated with EuB_6_@RGD‐K NPs+NIR‐III 1550 nm light irradiation, which is far longer than that for the untreated control group (18 d).

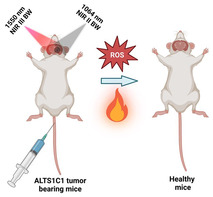

## 1 Introduction

Glioblastoma multiforme (GBM) is primarily referred as grade IV astrocytoma, which is fast‐growing and most aggressive fatal orthotopic brain tumor, originating from the glial cells of the central nervous system (CNS),^[^ ^1^, ^2^, ^3^ ^]^ and it is the predominant malignancy among other intracranial tumors. The standard treatment for GBM includes neurosurgical removal of the tumor, followed by chemotherapy and radiotherapy. However, the efficacy of these standard treatments is significantly hampered by various formidable obstacles presented by the aggressive nature of GBM multiforme. In particular, the diffusive and infiltrative characteristics of GBM make total resection nearly impossible during surgery. The intricate network of tumor cells infiltrating the adjacent tissues poses a significant challenge, limiting the success of surgical removal. Additionally, the presence of the blood‐brain‐barrier (BBB) further complicates the treatment, restricting the effective delivery of chemotherapeutic drugs to the tumor microenvironment (TME). Furthermore, the skull acts as a barrier, reducing the effective dose of radiation that reaches the TME during radiotherapy. Despite extensive surgery and concurrent chemotherapy and radiotherapy, the survival rates for GBM remain alarmingly low, at only 10–14 months as reported in a study in 2022.^[^ ^4^, ^5^ ^]^ Beyond the treatment efficacies, there are many other pre‐ and post‐surgical complications that include wrong‐side surgery, hemorrhage/hematoma complicated procedures, retention of foreign objects, iatrogenic stroke, meningitis, and toxic side effects experienced in the healthier regions of the brain due to chemotherapy and radiotherapy, which can significantly impact the quality of life for GBM patients.^[^ ^6^ ^]^ While the standard treatments provide initial benefits, the complex brain environment and infiltrative nature of the GBM multiforme allow only limited success and low survival rates. Concurrently, monitoring the progress of GBM treatment is also challenging because treatment‐related changes and progressing tumor lesions often exhibit similar characteristics that affect clinical decision‐making.^[^ ^7^ ^]^ The most important factor for the poor prognosis of GBM is its resistance to chemodrug‐induced apoptosis, the difficulty of drug‐crossing the BBB and X‐Ray beam passing through the skull.^[^ ^8^ ^]^ Therefore, it leaves a grand challenge to develop an innovative therapeutic strategy with precise diagnostic capabilities to cater to the clinical care of deeply‐seated GBM. To overcome these challenges, the field of light‐activatable nanotheranostics has emerged as a promising avenue, leveraging the unique properties of light to penetrate deeper into the brain tissues and combating GBM at the molecular level noninvasively. The use of near‐infrared (NIR) light, in particular, has garnered significant attention for its ability to penetrate deep into biological tissues with minimal absorption, allowing for non‐invasive activation of photoresponsive nanomaterials. The longer into the NIR wavelength region is, including NIR‐I (650–950 nm), NIR‐II (1000–1350 nm), and NIR‐III (1550–1800 nm) biological windows, the deeper the depth of tissue penetration^[^ ^4^ ^]^ and reduced photodamages to surrounding healthy cells will be. This not only enables targeted drug release within the tumor microenvironment but also facilitates an imaging modality to monitor therapeutic outcomes without the need for invasive procedures.

To this end, several NIR light‐activated theranostic interventions have been developed to target GBM. For example, Lee et al. have developed lanthanide‐doped upconversion nanoparticles (UCNPs) for image‐guided NIR‐I photothermal therapy (PTT). In particular, a continuous wave (CW) 808 nm laser for PTT and a pulsed wave (PW) 980 nm laser have been utilized for upconversion luminescence (UCL) imaging.^[^ ^9^ ^]^ Similarly, 808 nm light‐activated chlorin e6 (Ce6)‐loaded UCNPs were used for combinatorial PDT and CO therapy for the treatment of brain tumors. The major limitations of using UCNP‐based systems are: a) ultra‐low molar extinction coefficients in the NIR region; b) restriction in the choice of the excitation wavelength (only limited to 808 and 980 nm); c) requirement of very high laser power densities ranging from 1 to 10^3^ W cm^−2^; d) requirement of visible light photosensitizers; and e) indirect photosensitization process leading to very low overall singlet O_2_ quantum yields.^[^ ^10^, ^11^ ^]^ Another pioneering study has used NIR‐II 1064 nm laser for the activation of conjugated polymeric nanoparticles for PTT and photoacoustic (PA) imaging of GBM.^[^ ^4^ ^]^ In another study, Yin et al. Has utilized both 808 and 1064 nm lasers to stimulate PTT and PA imaging of deep‐seated GBM using perylene monoamine nanoparticles.^[^ ^12^ ^]^ While the aforementioned studies have demonstrated promising outcomes, there remains a notable gap in exploring the combination of PTT and PDT as well as a thorough investigation of the potential benefits offered by the NIR‐II and NIR‐III biological windows, particularly for distinctive imaging. These areas are particularly critical for addressing the challenges posed by the skull as a barrier in the treatment of GBM. Existing research utilizing 808 and 1064 nm lasers has laid a foundation, yet there is a compelling need to delve deeper into the distinct advantages offered by NIR‐II and NIR‐III for synergistic phototheranostics. This could further optimize treatment outcomes, considering their potential to minimize absorption by surrounding healthy brain tissues and enhance penetration through the skull, delineating the tumor clearly. Such advancements hold promise for significantly improving the efficacy and precision of therapeutic interventions for the challenging landscape of GBM treatment. We have summarized a brief literature overview of the development of phototheranotic agents that are capable to tackle deeply seated tumors (see **Table** **1**). Combination of photodynamic therapy with many other therapies, such as CO gas therapy and RNAi therapy, as well as imaging modalities were also developed for inhibition of tumor growths, metastasis, and recurrences.^[^ ^13^, ^14^, ^15^, ^16^, ^17^, ^18^, ^19^ ^]^

**Table 1:** Brief literature overview of the development of phototheranotic agents aiming to tackle deeply seated tumors.

S.No	Nanomaterial	Size [nm]	Targeting agent	Wavelength	In vivo tumor model	References
1	ANG‐IM NPs	80	Angiopep‐2 (LRP‐1)	808 nm (PTT), 980 nm (PDT) (NIR‐I & ‐II)	Orthotopic astrocytoma mice model	[46]
2	P1RGD NPs	60	cRGD (integrin)	1064 nm (NIR‐I)	Subcutaneous and orthotopic xenograft glioma mice model	[4]
3	MWCNTs	15	–	970 nm (NIR‐II)	–	[47]
4	ICG/AuNR@BCNP	150	Albumin (SPARC)	808 nm (NIR‐I)	Subcutaneous glioma mice model	[48]
5	UCNP‐KR‐LP	200	Lead peptide (integrin beta‐1)	980 nm (NIR‐II)	Xenograft MDA‐MB‐231 mice model	[49]
6	STPNs	150	Trastuzumab (HER‐2)	980 nm (NIR‐II)	Orthotopic GBC mice model	[50]
7	BPNS‐GNBP	80	–	808 nm (NIR‐I)	Orthotopic lung tumor mice model	[51]
8	iRGD‐rHDL/ICG NPs	90	iRGD (integrin)	808 nm (NIR‐I)	Subcutaneous 4T1 mice model	[52]
9	PFP/ICG/DOX@LIP nanodroplets	362	–	808 nm (NIR‐I)	Xenograft MDA‐MB‐231 mice model	[53]
10	AHZ NPs	90	–	1064 nm (NIR‐II)	Subcutaneous 4T1 mice model	[54]
11	GOF‐Lipo‐FA	200	Folic acid (Folate receptor)	808 nm (NIR‐I)	Subcutaneous 4T1 mice model	[55]
12	Nano‐BFF	140	–	808 and 1064 nm (NIR‐I & ‐II)	Orthotopic HCC mice model	[56]
13	EuB_6_@RGD‐K NPs	20	RGD‐K peptide (α_v_β_3_ integrin)	1550 nm (NIR‐III)	Murine brain tumors	Present study

**Scheme 1:**
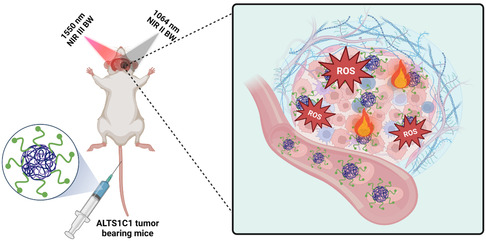
Schematic representation of the unprecedented in vivo phototherapy of ALTS1C1 tumor‐bearing mice in NIR‐II and NIR‐III biological windows using systemically injected EuB_6_@RGD‐K NPs.

In this study, we have engineered a tumor‐specific europium hexaboride (EuB_6_ NPs)‐based nanotheranostic agent with MR image‐guided NIR‐II (1064 nm) and NIR‐III (1550 nm) phototherapeutic capabilities to tackle deeply seated GBM. After surface modification with RGD‐K peptide, these EuB_6_ NPs were able to achieve molecular specificity toward ALTS1C1 glioblastom cancer cells which overexpress α_v_β_3_ integrin receptors. Further, EuB_6_@RGD‐K NPs are able to exert theranostic capabilities to effectively diagnose and combat difficult‐to‐treat orthotopic glioblastoma tumors using NIR‐II 1064 nm and NIR‐III 1550 nm combined photodynamic and photothermal therapy effects. With very large molar extinction coefficients in the NIR‐II and NIR‐III BWs, EuB_6_ NPs exhibit good photostability with very high photothermal conversion efficiency (*η*) (≈39.2% at NIR‐III 1550 nm light irradiation). To the best of our knowledge, for the first time, we show that EuB_6_ NPs can sensitize the formation of singlet O_2_ upon NIR‐II 1064 nm photoexcitation, and generation of hydroxyl radicals using NIR‐III 1550 nm light. We demonstrate that these EuB_6_@RGD‐K NPs could mediate NIR‐II (1064 nm) and NIR‐III (1550 nm) combined photodynamic and photothemal therapeutic effects on destructing tumor cells in both in vitro and in vivo models. The inclusion of targeted nanomaterials, such as EuB_6_@RGD‐K, can help eliminate invasiveness, achieve better precision, improve imaging contrast, and accelerate temperature rise. The non‐invasive nature of NIR light coupled with photosensitive nanomaterials presents a promising solution to the challenges associated with conventional treatment modalities. This approach allows for high spatio‐temporal treatment precision even for aggressive deeply‐seated GBM multiformes. Its non‐invasiveness mitigates the risk of damages to critical structures, thereby enhancing the safety profile of treatment. Moreover, it is a minimally invasive clinical procedure that is primarily adapted for ablation of intracranial tumors. Taken all together, our findings propose a novel long NIR‐activated strategy to improve the prognosis and quality of life of patients with GBM (**Scheme** **1**).

## 2 Results and Discussion

### 2.1 Synthesis and Characterization of EuB_6_@RGD‐K NPs

**Figure 1:**
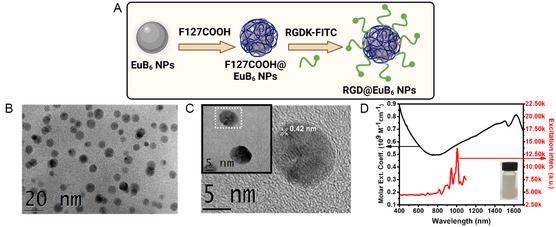
A) Schematic representation of the EuB_6_@RGD‐K NPs synthesis. B and C) represent the low‐ and high‐magnification TEM images of the as‐synthesized EuB_6_ NPs, respectively; D) UV‐visible‐NIR absorption spectrum (solid black trace) and excitation spectrum (solid red trace) for singlet oxygen phosphorescence emission (*λ*_em_ = 1268 nm) from photo‐excited EuB_6_ NPs. The inset shows the aqueous dispersion of EuB_6_ NPs.

The EuB_6_ NPs were synthesized by following a literature procedure with slight modification.^[^ ^20^ ^]^ Further, these nanoparticles were surface‐chelated with a biocompatible polymer, Pluornic F127‐COOH, followed by the conjugation of fluorescein isothiocyanate (FITC)‐labeled RGD‐K peptides to facilitate molecular specificity toward α_v_β_3_ integrin receptors overexpressed on the surface of ALTS1C1 glioblastoma cancer cells (see **Figure** **1**A). The transmission electron microscopy (TEM) images clearly show that as‐synthesized EuB_6_ NPs exhibited a good crystallanity and spherical morphology with an interplanar *d*‐spacing of ≈0.42 nm (Figure 1B,C and S1, Supporting Information). The UV‐visible‐NIR absorption spectrum of EuB_6_ NPs displays a broad absorption up to 1800 nm, covering the NIR‐I (650–950 nm), NIR‐II (1000–1350 nm), and NIR‐III (1550–1800 nm) biological windows (Figure 1D). The molar extinction coefficients of EuB_6_ NPs were determined to be 0.6 × 10^9^ M^−1^ cm^−1^ at 1064 nm (NIR‐II BW) and 0.75 × 10^9^ M^−1^ cm^−1^ at 1550 nm (NIR‐III BW), respectively, which are at least 5–7 orders higher than that of the convention organic photosensitizer dyes, and 1–2 orders higher than those of other metallic nanoparticles.

The powder X‐ray diffraction (PXRD) spectrum shown in Figure S2, Supporting Information, clearly reveals the presence of europium hexaboride, which is in good agreement with the JCPDS card no. 40‐1308. We have also investigated the magnetic properties of as synthesized EuB_6_ NPs using SQUID (superconducting quantum interference device) analysis. As shown in Figure S3, Supporting Information, the EuB_6_ NPs displayed weak magnetic moment at both 5 and 298 K temperatures. In the clinical settings, the magnetic property provides an opportunity for EuB_6_ NPs to serve as a MRI contrast agent for in‐vivo monitoring the therapeutic outcomes. To introduce tumor‐targeting abilities toward glioblastoma tumors, EuB_6_ NPs were functionalized with FITC‐labeled RGD‐K peptides. First, to achieve good aqueous dispersibility, EuB_6_ NPs were chelated with a biocompatible polymer, Pluronic F127‐COOH, using a previously reported procedure.^[^ ^21^, ^22^, ^23^ ^]^ Subsequently, F127‐COOH coated EuB_6_ NPs were conjugated with FITC‐labeled RGD‐K peptide via EDC‐NHS coupling chemistry. As shown in Figure S4, Supporting Information, the FTIR spectra revealed the presence of the characteristic peaks of the carbonyl group (C=O stretching) at 1726 cm^−1^ and the hydroxyl group (O—H stretching) at 2883 cm^−1^ for F127‐COOH‐chelated EuB_6_ NPs. Furthermore, FITC‐labeled RGD‐K peptide conjugation was confirmed by the presence of two characteristic amide stretching peaks at 1536 and 1642 cm^−1^. The presence of FITC‐labeled RGD‐K peptide on the surface of EuB_6_ NPs was also measured quantitatively using a bicinchoninic acid (BCA) assay.

**Figure 2:**
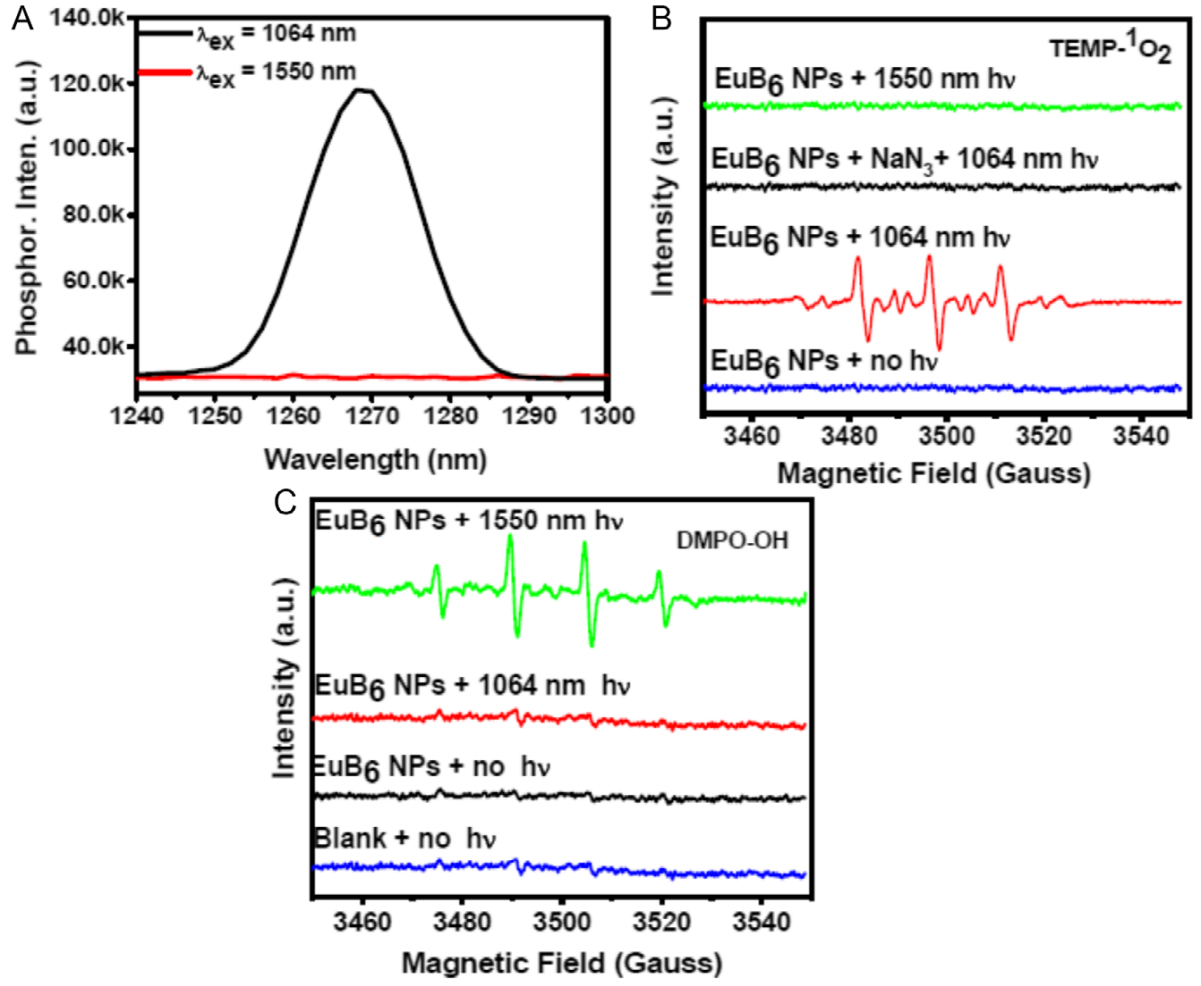
A) Singlet O_2_ phosphorescence emission spectra of EuB_6_ NPs at photoexcitation wavelengths of 1064 and 1550 nm. A long pass filter of 1000 nm was used to remove stray and scattered light from the excitation wavelength. B and C) show the EPR spectra of TEMP‐^1^O_2_ and DMPO‐OH adducts for EuB_6_ NPs under dark and light irradiation conditions, respectively.

The UV‐visible absorption spectrum of EuB_6_@RGD‐K NPs revealed two distinct peaks centered at 290 and 489 nm, which corresponded to the tyrosine residues of the RGD‐K peptides and FITC fluorescent marker, respectively. Subsequently, at a photo‐excitation wavelength of 490 nm, the fluorescence spectra of the FITC‐labeled EuB_6_@RGD‐K NPs revealed an emission maximum at 525 nm (Figure S5, Supporting Information). The hydrodynamic diameters of EuB_6_ NPs and EuB_6_@RGD‐K NPs measured using dynamic light scattering (DLS) were 11.0 ± 3 and 18.0 ± 5 nm, respectively. Furthermore, the zeta‐potential values of EuB_6_ NPs, F127‐COOH chelated EuB_6_ NPs, and EuB_6_@RGD‐K NPs indicate net charges of 5.0, −17.0, and −6.0 mV, respectively (Figure S6, Supporting Information). Owing to their superior light‐absorbing capabilities across NIR‐I, ‐II, and ‐III biological windows, EuB_6_ NPs can sensitize the formation of singlet oxygen (^1^O_2_) upon NIR‐II 1064 nm photo‐excitation (see red trace in Figure 1D and **2**A). Subsequently, upon the addition of a singlet O_2_ specific quencher, namely, sodium azide, the singlet oxygen phosphorescence emission was significantly quenched (see Figure S7, Supporting Information). The singlet O_2_ quantum yield of EuB_6_ NPs at NIR‐II 1064 nm photoexcitation was determined to be 0.187 (see Supporting Information for details). Furthermore, the photo‐induced generation of reactive oxygen species (ROS), such as singlet O_2_ and hydroxyl radicals (OH) was examined by electron paramagnetic resonance (EPR) spectroscopy. As shown in Figure 2B,C, upon photoexcitation at 1064 nm, distinct singlet O_2_ signals originated from the TEMP‐^1^O_2_ adduct. In the presence of sodium azide as a specific quencher for singlet O_2_, there were no detectable EPR signals, confirming the identity of the singlet O_2_. Firstly, to detect the presence of singlet O_2_, 2,2,6,6,‐tetramethyl‐4‐piperidone (TEMP) was used as a spin‐trapping reagent (Figure 2B). The formation of hydroxyl radicals was also examined using 5,5‐dimethyl‐1‐pyrroline N‐oxide (DMPO) as a hydroxyl radical spin trapping reagent. As shown in the Figure 2C, the characteristic 1:2:2:1 pattern of the DMPO‐OH adduct was observed upon 1550 nm light irradiation of EuB_6_ NPs‐containing aqueous solution (see the green curve in Figure 2C), but not by 1064 nm light irradiation. The mechanism of generation of hydroxyl radicals by 1550 nm NIR light irradiation of EuB_6_ NPs is probably similar to that for LaB_6_ NPs, since rare earth metal hexaborides might share some common or similar chemical properties.^[^ ^24^ ^]^ In brief, water molecules probably undergo dissociative adsorption on the surface of EuB_6_ NP's surface to form hydorxide anion nearby the Eu^3+^ cations and proton cation nearby the B_6_^3−^ cluster anions, similar to that for LaB_6_ NPs.^[^ ^25^, ^26^ ^]^ Upon 1550 nm photo‐excitation, the surface‐bound hydoxide anion was oxidized by the holes (probably the Eu^4+^ cations) to generate hydroxyl radicals, as that observed in the LaB_6_ system.^[^ ^24^ ^]^ The detail mechanism for the formation of hydroxyl radicals from 1550 nm NIR light irradiation of EuB_6_ NPs requires detail studies in the future.

To sensitize the formation of singlet oxygen, two factors must be fulfilled, namely: a) the excited state energy of the donor must be larger than the bandgap energy of singlet oxygen, that is 0.98 eV; and b) the excited state of the energy donor must be in the triplet excited state. The photon energy of 1064 nm (i.e., 1.165 eV) is higher than that of the singlet oxygen, whereas the photon energy of 1550 nm (i.e., 0.8 eV) is lower than that of the singlet oxygen. Therefore, it is not possible to use 1550 nm one‐photon excitation of any photosensitizers to sensitize the formation of singlet oxygen. Most probably, the wavefunction of triplet plasmonic excited states of EuB_6_ NPs upon 1064 nm has good similarity or overlap with that of triplet ground state molecular oxygen, so that energy transfer from triplet plasmonic excited states of EuB_6_ NPs to molecular oxygen could occur, leading to the formation of singlet oxygen. The details for the energy transfer from triplet plasmonic excited states of EuB_6_ NPs to molecular oxygen are still not clear at this stage and require further experimental and theoretic studies in the future.

Taken together, these results corroborate that EuB_6_ NPs can sensitize the formation of singlet O_2_ at a 1064 nm photoexcitation wavelength (NIR‐II BW) and the formation of hydroxyl radicals upon 1550 nm photo‐excitation. Further, the photothermal temperature rise for EuB_6_ NPs using NIR‐II 1064 nm and NIR‐III 1550 nm photo‐excitation wavelengths (0.3 W cm^−2^; 10 min) were observed to be 44.7 and 54.7 °C, respectively (see Figure S8A, Supporting Information). Such results clearly indicate that photo‐irradiation of EuB_6_ NPs by both NIR‐II 1064 nm and NIR‐III 1550 nm generates a combination of ROS‐based photodynamic and photothermal effects. Accompany of photodynamic effect (i.e., generation of ROS) by phototheraml effect is not surprising, since non‐radiative decay process of photo‐excited states always occurs for nearly all kinds of light‐absorbing molecules and nanomaterials. Notice that there are no noticeable alterations in the morphologies and uv‐vis‐NIR absorption profiles of EuB_6_ NPs before and after photo‐irradiation using NIR‐III 1550 nm light (Figure S8B, Supporting Information). The photothermal conversion efficiency of EuB_6_ NPs using NIR‐III 1550 nm excitation wavelength was measured to be ≈39.2%, which is 1.5 times higher than the literature reported copper selenide nanoparticles (≈22% at NIR‐I 808 nm) and copper sulphide nanoparticles (≈25.7% at NIR‐II: 980 nm),^[^ ^27^, ^28^ ^]^ respectively (Figure S9, Supporting Information). The photothermal conversion efficiency of EuB_6_ NPs upon 1064 nm NIR light irradiation was also determined to be 31.4%, which is different from and slightly lower than the value of 39.2% photothermal conversion efficiency of EuB_6_ NPs upon 1550 nm NIR light irradiation (Figure S9, Supporting Information). Such an excitation wavelength‐dependent photothermal conversion efficiency from the same nanomaterial was also observed before from gold nanoparticles.^[^ ^29^ ^]^ The detailed mechanism responsible for such an excitation wavelength‐dependent photothermal conversion efficiency is still not clear at this stage. One possible reason could be that different excitation wavelengths result in the excitation of EuB_6_ NPs to different plasmonic excited states. In the literature, it is known that surface plasmon resonance states are localized and do not conjugate or interact with each other. Likewise, the plasmonic excited states are also localized and do not interact with each other. In such a case, different excitation wavelengths could excite EuB_6_ NPs to different localized plasmonic excited states, from which all plasmonic excited states have their own photophysical properties and photothermal conversion/relaxation efficiencies.

Further, we have investigated the percentages of light penetration (relative to the original laser intensity before passing through the skull) through the mice skull using NIR‐II 1064 nm and NIR‐III 1550 nm irradiation conditions. As shown in Figure S10A, Supporting Information, the percentage of light penetration through the mice skull using NIR‐II 1064 nm and NIR‐III 1550 nm irradiation conditions (power density: 0.5 W cm^−2^) are 67% and 80%, respectively. In order to determine the tissue penetration depths using NIR‐II 1064 nm and NIR‐III 1550 nm lights, a pork tissue with variable thicknesses has been used at a power density of 0.5 W cm^−2^. It is clear that, for 5 mm pork tissue thickness, the light intensity penetration is ≈1.6 times higher for NIR‐III 1550 nm irradiation when compared to that of NIR‐II 1064 nm irradiation (Figure S10B, Supporting Information).

### 2.2 In Vitro NIR‐II/‐III Combined Photodynamic and Photothermal Effects Mediated by EuB_6_@RGD‐K NPs.

In order to achieve excellent phototherapeutic effects on killing cancer cells or destroying solid tumors, it is first essential to have an ultrahigh intracellular uptake of a nanomedicine. To this end, we first evaluated the cellular uptake of EuB_6_@RGD‐K NPs in ALTS1C1 cancer cells by measuring the characteristic green fluorescence originating from the RGD‐K peptide conjugated onto the surface of EuB_6_ NPs using confocal laser scanning microscopy and flow cytometry.

**Figure 3:**
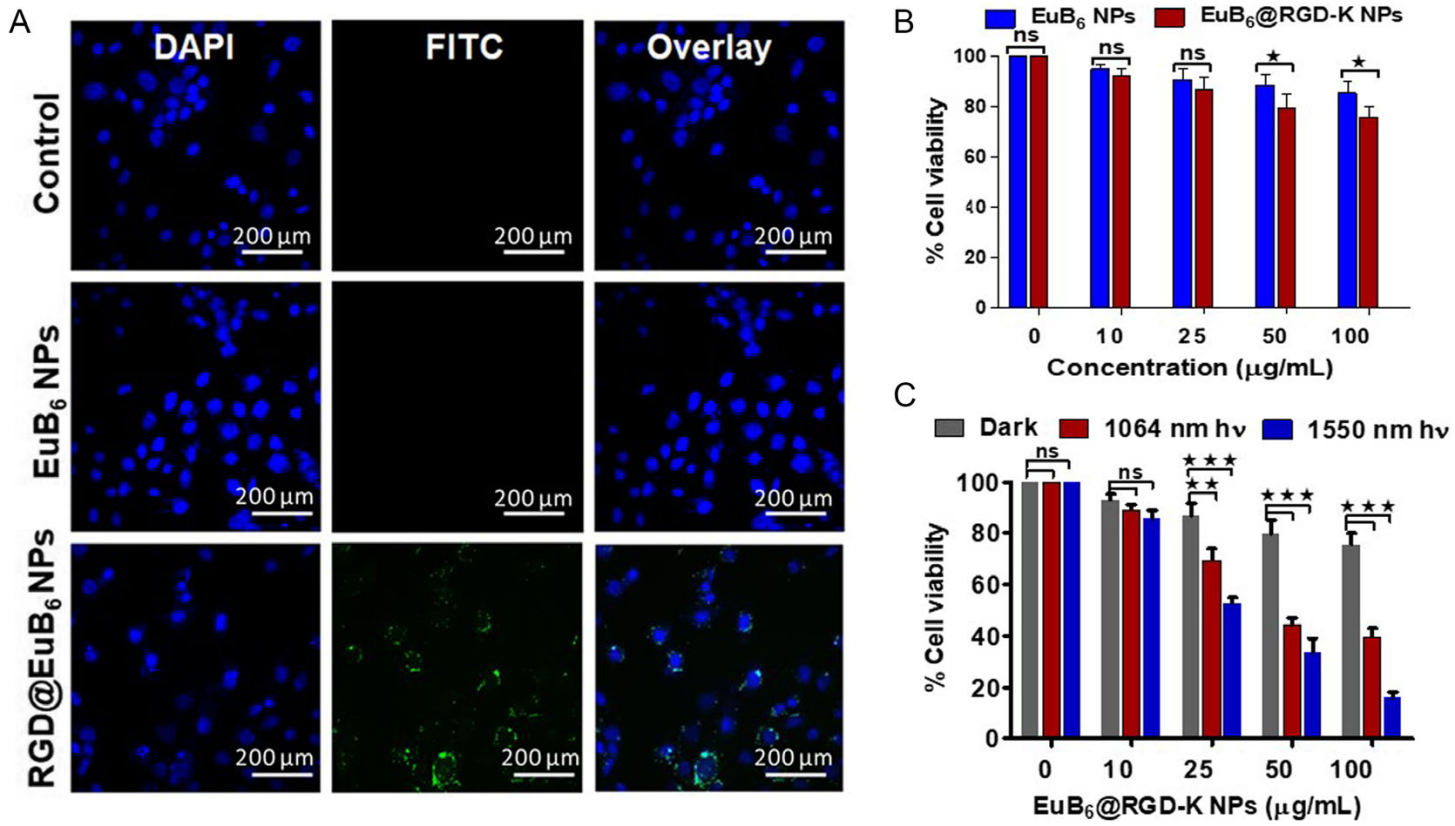
A) Cellular uptake of EuB_6_ NPs‐(without surface‐modified with RGD‐K peptides) and EuB_6_@RGD‐K NPs‐internalized ALTS1C1 cells monitored using confocal laser scanning microscopy (CLSM). B and C) represent the percentages of cell viabilities of ALTS1C1 cancer cells incubated with different concentrations of EuB_6_ NPs and EuB_6_@RGD‐K NPs, respectively, under dark and photo‐irradiation (1064 and 1550 nm lasers) conditions. The statistically significant differences are indicated as *p < 0.05, **p < 0.01, and ***p < 0.001. The scale bar is 200 μm.

The confocal images of the EuB_6_@RGD‐K NPs‐internalized ALTS1C1 cells clearly showed significant green fluorescence emission that was evenly distributed in the cytoplasm region without entering into the nucleus (**Figure** **3**). To determine the molecular specificity of EuB_6_@RGD‐K NPs internalized ALTS1C1 cells, we measured the intracellular uptake using flow cytometry. As expected, the targeted EuB_6_@RGD‐K NPs internalized ALTS1C1 cancer cells showed ≈3.0‐fold higher FITC fluorescence intensity signals when compared to the No NPs‐fed control group (see Figure S11, Supporting Information). To examine the cytotoxicity of the EuB_6_ NPs and EuB_6_@RGD‐K NPs‐internalized ALTS1C1 cells, an MTT (3‐(4,5‐dimethylthiazolyl)‐2)‐2,5‐diphenyltetrazolium bromide) cell viability assay was performed. The cellular viabilities in Figure 3B clearly show that the EuB_6_ NPs and EuB_6_@RGD‐K NPs exhibited concentration‐dependent cytotoxicity behavior. The half‐maximal inhibitory concentration (IC_50_) values of the EuB_6_ NPs and EuB_6_@RGD‐K NPs were >100 μg/mL. To demonstrate that these multifunctional EuB_6_ nanoparticles can act as excellent phototherapeutic agents for killing the cancer cells, EuB_6_@RGD‐K NPs internalized ALTS1C1 cells were exposed to both NIR‐II 1064 nm (0.3 W cm^−2^, 10 min) and NIR‐III 1550 nm (0.3 W cm^−2^, 7 min) laser irradiation, respectively. The laser irradiation time at different wavelengths was adjusted to compensate for the differences in the extinction coefficients so that the amounts of photons being absorbed by the EuB_6_@RGD‐K NPs will be the same and the phototherapy effects at different wavelengths can be fairly compared. As shown in Figure 3C, upon photo‐irradiation using NIR‐II 1064 nm or NIR‐III 1550 nm lights, significant cellular deaths were observed. For example, at a EuB_6_@RGD‐K NPs dose of 100 μg mL^−1^ concentration, the cellular viabilities being ≈39.5% and 16% for 1064 and 1550 nm light irradiation, respectively. Subsequently, the Calcein AM and propidium iodide fluorescence microscopy images reveal that EuB_6_@RGD‐K NPs internalized ALTS1C1 cells and exposed to NIR‐II 1064 nm or NIR‐III 1550 nm lights exhibit pronounced cellular deaths (see Figure S12, Supporting Information). The results in Figure 3C clearly indicate that the 1550 nm induced photo‐cytotoxicity is much larger than that from the 1064 nm light irradiation. As shown in Figure 2, 1064 nm light irradiation of EuB_6_ NPs can sensitize the formation of singlet O_2_, as evidenced by the phosphorescence emission centered at 1270 nm, as well as from EPR experiments. Whereas the NIR‐III 1550 nm light irradiation of EuB_6_ NPs induces the formation of hydroxyl radicals as well as hyperthermia. The photon energy of 1550 nm is lower than the bandgap energy (≈0.98 eV, equivalent to a photon wavelength of 1265 nm) of singlet oxygen. Therefore, it is not possible for 1550 nm light‐excited EuB6 to transfer excited state energy to molecular O_2_ and generate singlet O_2_. Further, to provide an in‐depth understanding of the contributions of photothermal effects on the cell deaths of the EuB_6_@RGD‐K NPs‐internalized ALTS1C1 cells upon NIR‐II 1064 nm and NIR‐III 1550 nm light irradiations, heat shock protein (HSP70) expression levels were monitored using flow cytometry. As shown in Figure S13, Supporting Information, the HSP70 expression levels were significantly higher for the EuB_6_@RGD‐K NPs‐internalized ALTS1C1 cells exposed to NIR‐III 1550 nm light, when compared to that of the NIR‐II 1064 nm light as well as in the dark. In the literature, it is known that a small amount of heat shock proteins could protect other proteins/enzymes from misfolding under heat stress, and thus protecting cells from heat stress‐induced apoptosis. However, it is also known that overexpression or a large amount of heat shock proteins will trigger the cellular apoptosis cascades and induce cell deaths.^[^ ^30^, ^31^ ^]^ Overall, our in vitro results suggest that EuB_6_@RGD‐K NPs can mediate combined photodynamic and photothermal therapy effects upon both NIR‐II (1064 nm) and NIR‐III (1550 nm) light irradiation on effective killing of ALTS1C1 glioblastoma cancer cells.

### 2.3 In Vivo NIR‐II/‐III Photodynamic Therapeutic Effects Mediated by EuB_6_@RGD‐K NPs

**Figure 4:**
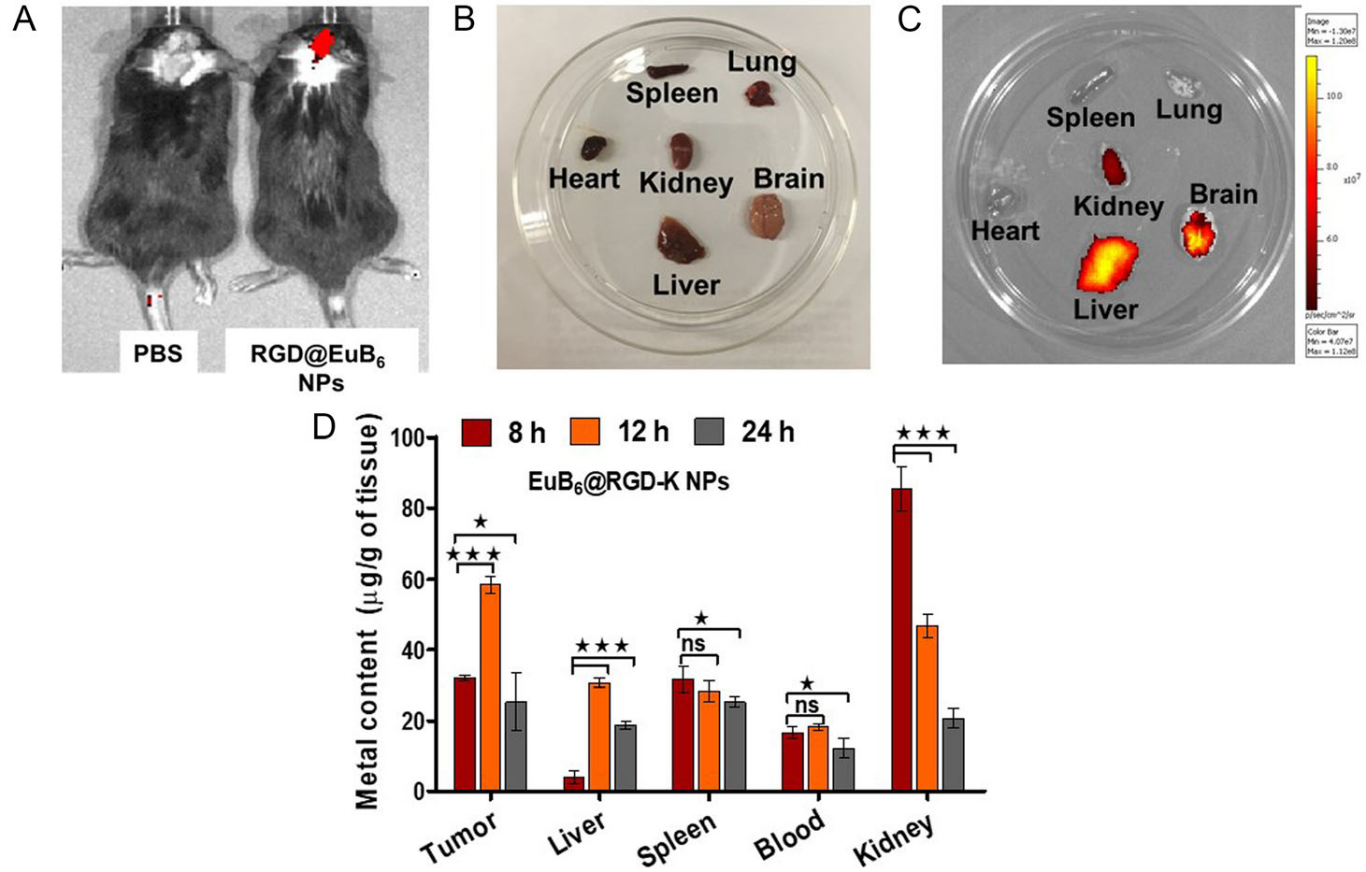
A) In vivo whole body animal imaging of PBS‐ and Cy5.5‐tagged EuB_6_@RGD‐K NPs‐injected mice implanted with glioblastoma tumors (*λ*_ex_ = 615–665 nm; *λ*_ex_ = 695–770 nm). B and C) represent the ex vivo white light and fluorescence images of major organs such as, liver, spleen, heart, lung, kidney, and brain from the mice intravenously injected with PBS and Cy5.5 tagged EuB_6_@RGD‐K NPs, respectively. D) In vivo biodistribution of EuB_6_@RGD‐K NPs in various organs after 8, 12, and 24 h post‐intravenous injection in tumor implanted mice. The statistically significant differences are indicated as *p < 0.05, **p < 0.01, and ***p < 0.001.

We have performed in vivo experiments to evaluate the phototheranostic capabilities of EuB_6_@RGD‐K NPs to mediate NIR‐II NmPDT and NIR III NmPTT effects against deeply seated orthotopic glioblastoma tumors. To this end, ALTS1C1 cells were intracranially implanted into the brain region through surgery in C57BL/6J mice. Post‐tumor implantation, both PBS and EuB_6_@RGD‐K NPs were administered systemically and the whole body animal imaging was performed. For the purpose of in vivo whole body animal imaging, EuB_6_@RGD‐K NPs were tagged with the long‐red Cy5.5 fluorphore. The photoluminescence spectra of Cy5.5‐tagged EuB_6_@RGD‐K NPs revealed a significant fluorescence emission between 700 and 820 nm upon using 670 nm excitation (see Figure S14, Supporting Information). As shown in **Figure** **4**A, the in vivo whole animal imaging reveals that the Cy5.5‐tagged EuB_6_@RGD‐K NPs accumulated at the brain site at 14 h post intravenous injection. Subsequently, all the major organs from the mice, such as liver, kidney, heart, lung, spleen, and brain were dissected and imaging was performed. The ex vivo images for Cy5.5‐tagged EuB_6_@RGD‐K NPs injected mice clearly reveal intense fluorescence signals from liver, kidney, and brain. In contrast, PBS‐injected control mice did not exhibit any noticeable fluorescence emission signals (see Figure 4B,C). However, the major organs of reticuloendothelial system (RES), such as liver and spleen also showed slightly high levels of EuB_6_@RGD‐K NPs accumulation. Most likely, the Kuppfer cells in the liver and splenic macrophages are responsible for the clearance EuB_6_@RGD‐K NPs from the blood stream. Furthermore, we have also investigated the in vivo biodistribution of the EuB_6_@RGD‐K NPs at 8, 12, and 24 h post‐injection using inductively coupled mass spectrometry (ICP‐MS). In Figure 4D, higher levels of europium of ≈58 μg g^−1^ of tissue at the tumor site were observed at 12 h post injection when compared to that of 8 and 24 h post‐injection. In constrast, the europium levels in the blood was ≈18.5 μg g^−1^ at 12 h post‐injection, which is ≈3.0 times lower than the levels in the tumor site. Hepatic clearance and renal clearance are two major in vivo clearance pathways for “foreign” nanomaterials.^[^ ^32^ ^]^ Nanoparticles with sizes smaller than 10 nm will be cleared by kidney. As shown in the Figure 3, the dark cytotoxicities of EuB_6_ NPs at various doses are very low. In the absence of photo‐irradiation, no ROS will be generated by EuB_6_ NPs. Notice that to treat and kill tumor cells, photo irradiation was exerted only to the tumor site, but not to the kidney. Therefore, it is not expected to have inflammatory damages in the kidney in the absence of photo irradiation. Figure S15, Supporting Information, shows the H&E staining for different organs after treatment and we did not observe any inflammatory damages to kidney, since no photo irradiation was exerted at the kidney site.

**Figure 5:**
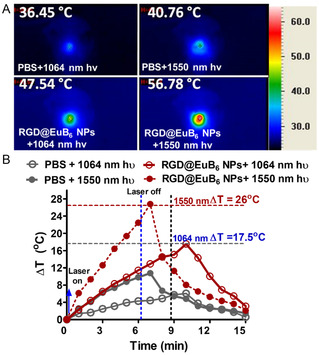
A) In vivo photothermal images of mice injected with PBS and EuB_6_@RGD‐K NPs in ALTS1C1 tumor bearing mice and exposed to both NIR‐II 1064 nm and NIR‐III 1550 nm lasers. The values indicated are the final temperatures for mice exposed to different wavelength lasers. B) The change in temperature rise (Δ*T*) profiles were plotted as a function of irradiation times for the mice injected with PBS and EuB_6_@RGD‐K NPs exposed to 1064 and 1550 nm lasers.

**Figure 6:**
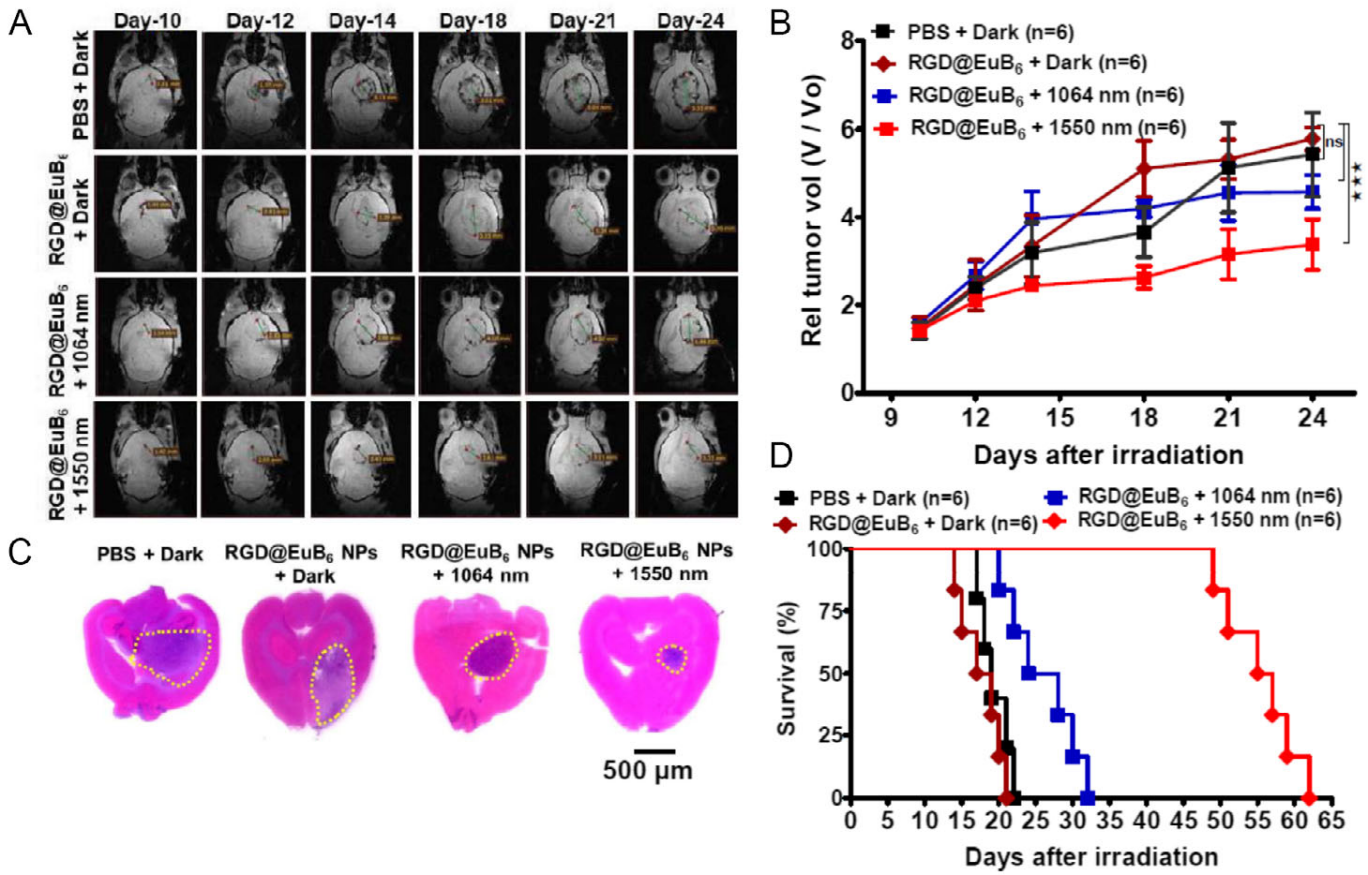
A) *T*_1_ weighted MR images of brain sections from PBS, EuB_6_@RGD‐K NPs‐injected mice exposed to both NIR‐II 1064 nm and NIR‐III 1550 nm lasers as a function of time during the phototherapy. B) Tumor growth curves monitored for different treatment groups using MR imaging as a function of days after tumor implantation. C) Hematoxylin and eosin (H&E) staining for whole brain sections at 10 μm depth. D) Survival rate plots of the mice injected with PBS, EuB_6_@RGD‐K NPs exposed to different lasers. The statistically significant differences are indicated as *p < 0.05, **p < 0.01, and ***p < 0.001.

Overall, the in vivo biodistribution experiments revealed that presumably 12 h could be an optimal time point for performing the phototherapy. As shown in **Figure** **5**, the photo‐irradiation of EuB_6_@RGD‐K NPs injected ALTS1C1 glioblastoma brain tumor implanted mice causes a significant temperature rise, with a change in temperature (Δ*T*) of 17.5 and 26 °C, for NIR‐II 1064 nm and NIR‐III 1550 nm light irradiation, respectively. It has to be noted that the temperature rises at 1550 nm light irradiation is significantly higher than that of 1064 nm light irradiation. As a proof‐of‐concept study, we have carried out in vivo phototherapy experiments to combat deeply seated orthotopic glioblastoma brain tumors in mice. The ALTS1C1 glioblastoma brain tumors were implanted in C57BL/6J mice via intracranial injection at day 0. Subsequently, one group of mice were injected with PBS and another group of mice were injected with EuB_6_@RGD‐K NPs (50 mg kg^−1^) without any photo‐irradiation. The remaining two groups of mice were intravenously injected with EuB_6_@RGD‐K NPs and subjected to photo‐irradiation using NIR‐II 1064 nm laser (0.3 W cm^−2^; 10 min) and NIR‐III 1550 nm laser (0.3 W cm^−2^; 7 min), respectively. The tumor growth measurements for all the animal groups at days 10, 12, 14, 18, 21, and 24 were monitored using MR imaging. The intrinsic magnetic property of EuB_6_ NPs has been utilized to demonstrate the MR imaging capabilities (see Figure S3, Supporting Information). As shown in **Figure** **6**A, the mice treated with PBS in the dark showed an increase in the tumor size from 1.41 to 5.35 mm, whereas the mice treated with EuB_6_@RGD‐K NPs showed an increase in the tumor size from 1.45 to 5.96 mm, respectively. However, there is a significant reduction in the tumor sizes for the mice treated with EuB_6_@RGD‐K NPs and exposed with both NIR‐II 1064 nm and NIR‐III 1550 nm lasers (1.54 to 4.44 mm for NIR‐I 1064 nm; and 1.42 to 3.33 mm for NIR‐III 1550 nm). In Figure 6B, the relative tumor volumes of all the treatment groups were measured using MR imaging. The relative tumor volumes obtained for the EuB_6_@RGD‐K NPs treated and exposed to NIR‐III 1550 nm light irradiation indicated a superior delay in the tumor growth when compared to the other treatment groups (see the red trace in Figure 6B). During the course of therapy, the mice were euthanized on day 20^th^ and brain, kidney, liver, and spleen sections were removed and subjected to Hematoxylin and Eosin staining. The brain tissue sections from various treatment groups were examined and shown in Figure 6C, in which, the yellow circled regions indicate the relative tumor sizes at day 20^th^. Overall, it is very clear that the mice treated with EuB_6_@RGD‐K NPs and exposed to NIR‐III 1550 nm light irradiation showed relatively smaller tumor sizes as compared to all the mice treated with either PBS and EuB_6_@RGD‐K NPs (without light irradiation), and EuB_6_@RGD‐K NPs exposed to NIR‐II 1064 nm light irradiation. In addition, the kidney, liver and spleen histological sections for all the treatment groups did not show any noticeable necrosis (see Figure S15, Supporting Information). As shown in Figure S16, Supporting Information, the body weights in the mice did not show any noticeable drop for all the treatment groups. The average half‐life of 55 d for mice treated with EuB_6_@RGD‐K NPs and exposed to NIR‐III 1550 nm light irradiation is far higher than that of EuB_6_@RGD‐K NPs exposed to NIR‐II 1064 nm light irradiation (25 d), PBS‐treated mice (20 d), and EuB_6_@RGD‐K NPs treated mice (18 d) (see Figure 6D). Blood and lipid profile tests were performed for EuB_6_@RGD‐K NPs treated and non‐treated normal mice to determine the toxicity effects after 20 d oftreatment on different organ functions (liver, kidney, and spleen). The results of blood analysis clearly indicate no obvious changes between the treated group and control group as shown in Figure S17, Supporting Information. The long‐term toxicity studies using EuB_6_@RGD‐K NPs is an important concern for potential clinical translation. However, it require detailed investigations, which deserves a separate study in the future. Taken all together, these results suggest the successful destruction of “difficult‐to‐treat” complicated murine brain tumors using the combined NIR‐III (1550 nm) photodynamic and phototheraml therapies. To the best of our knowledge, this work is the first demonstration of destructing murine brain tumors via multi‐functional tumor‐specific europium hexaboride based nanotheranostic agent to mediate MR imaging‐guided NIR‐III phototherapy.

In the literature, several classes of nanomaterials have been demonstrated as phototheranostic agents in which both diagnostic and therapeutic modalities can be integrated into a single multi‐functional system to tackle various types of cancers. However, so far, these nanomaterials are capable of being activated by NIR light in the NIR‐I and ‐II biological windows.^[^ ^33^, ^34^, ^35^, ^36^, ^37^, ^38^, ^39^, ^40^ ^]^ However, it has to be noted that the nanomaterials that are activatable using NIR‐III light are very rare. Previously, we have developed a versatile plasmonic CuO/Cu_2_O truncated nanocubes‐based theranostic nanomedicine as trimodal image‐guided photothermal agents to kill multi‐drug resistant lung tumors using NIR‐III 1550 nm laser irradiation.^[^ ^41^ ^]^ However, plasmonic nanomaterials are unstable and prone to undergo photothermal reshaping under laser irradiation conditions.^[^ ^42^ ^]^ Therefore, there is a strong need to develop robust and stable NIR‐III phototherapeutic agents to kill “difficult‐to‐treat” tumors. To the best of our knowledge, the current work represents the first demonstration of a tumor‐specific europium hexaboride‐based theranostic nanomedicine capable of activation using NIR‐II and ‐III light to tackle “difficult‐to‐treat” murine glioblastoma tumors via MR image‐guided phototherapy. There are several hurdles for the nanomaterials to tackle “difficult‐to‐treat” murine glioblastoma tumors. For an effective brain tumor therapy, it is important to develop nanomaterials: i) with excellent molecular specificities or targeting the diseased site; ii) capable to cross BBB; iii) can offer deeper tissue penetration through the skull; iv) broad and extendable light absorbing capabilities, especially in the NIR‐III biological window; v) negligible cytotoxicity and non‐immunogenicity; vi) prolonged circulation half‐lives or enhanced pharmacokinetic/pharmacodynamic properties; and vii) exert an imaging modality which can help to monitor the therapeutic outcomes. In this regard, the current EuB_6_@RGD‐K NPs‐based theranostic system has several unique features which were never reported in the literature: 1) EuB_6_ NPs are of sub‐100 nm and have a broad and extendable absorption covering NIR‐I, ‐II and ‐III BWs (see Figure 1D); 2) The molar extinction coefficients of EuB_6_ NPs were determined to be 0.6 × 10^9^ M^−1^ cm^−1^ @ 1064 nm wavelength (NIR II BW) and 0.75 × 10^9^ M^−1^ cm^−1^ @ 1550 nm wavelength (NIR‐III BW), respectively, which are atleast 5–7 orders higher than that of the convention organic photosensitizer dyes, and 1–2 orders higher than those of other metallic nanoparticles; 3) The photothermal conversion efficiency of EuB_6_ NPs using NIR‐III 1550 nm excitation wavelength is ≈39.2%, which is 1.5 times higher than the literature reported copper selenide nanoparticles (≈22% at NIR‐I 808 nm) and copper sulphide nanoparticles (≈25.7% at NIR‐II: 980 nm),^[^ ^27^, ^28^ ^]^ respectively; 4) EuB_6_ NPs are capable to sensitize the formation of singlet O_2_ upon NIR‐II (1064 nm) photoexcitation as well as formation of hydroxyl radical upon NIR‐III 1550 nm photo‐excitation; 5) EuB_6_@RGD‐K NPs mediate excellent combined NIR‐III photodynamic and photothermal effects on killing ALTS1C1 glioblastoma cells; 6) The EuB_6_@RGD‐K NPs can serve as an excellent fluorescence and MR imaging agents to facilitate image‐guided phototherapy; 7) Prolonged circulation half‐lives with superior pharmacokinetic/pharmacodynamic capabilities; and 8) minimal toxicity and non‐immunogenicity. With all such unique features, these multi‐functional EuB_6_ NPs have a great potential to serve as a superior theranostic agent to tackle deeply seated tumors. Not just limited to tumors, these classes of nanoparticles can also be tailored to tackle other vascular and neurodegenerative disorders as well as other complicated diseases and cancers are capable of crossing all potential barriers involved in the bench‐to‐bed side translation.

During the review process, one reviewer requested us to measure and confirm the generation of singlet oxygen and hydroxyl radicals from EuB_6_@RGD‐K NPs‐internalized ALTS1C1 glioblastoma cancer cells. As shown in the supporting Figure S20, Supporting Information, the fluorescence intensity levels of SOSG exhibited ≈4.0‐fold higher for 1064 nm light irradiation of EuB_6_@RGD‐K NPs compared to the dark control group The SOSG fluorescence intensity levels for the 1550 nm light irradiation group are similar to those for the dark control group, which is expected since the photon energy (0.8 eV) of 1550 nm NIR light is lower than that (0.98 eV) required for the sensitization of singlet oxygen and thus formation of singlet oxygen is not possible from EuB_6_@RGD‐K NPs upon 1550 nm light irradiation. The ROS fluorescence levels of EuB_6_@RGD‐K NPs‐internalized ALTS1C1 cancer cells exhibited ≈3.0 fold higher for both 1064 and 1550 nm compared to dark. These results clearly show that EuB_6_@RGD‐K NPs are able to generate both ^1^O_2_ and hydroxyl radicals inside cancer cells.

## 3 Conclusions

In summary, we have successfully engineered a tumor‐specific europium hexaboride (EuB_6_ NPs)‐based nanotheranostic agent with MR image‐guided NIR‐II (1064 nm) and NIR‐III (1550 nm) combined photodynamic and photothermal therapies to tackle deeply seated glioblastoma tumors. After surface‐modification using RGD‐K peptide, these EuB_6_ NPs were able to achieve molecular specificity toward ALTS1C1 glioblastoma cancer cells, which overexpress α_v_β_3_ integrin receptors. Further, EuB_6_@RGD‐K NPs are able to exert theranostic capabilities to effectively diagnose and combat difficult‐to‐treat orthotopic glioblastoma tumors using NIR‐III 1550 nm combined photodynamic and photothermal therapy effects. The molar extinction coefficients of EuB_6_ NPs were determined to be 0.6 × 10^9^ M^−1^ cm^−1^@1064 nm wavelength (NIR‐II BW) and 0.75 × 10^9^ M^−1^ cm^−1^@1550 nm wavelength (NIR‐III BW), respectively, which are at least 5–7 orders higher than that of the convention organic photosensitizer dyes, and 1–2 orders higher than those of other metallic nanoparticles. The EuB_6_ NPs exhibit good photostability with very high photothermal conversion efficiency (*η*) (≈39.2% @ NIR‐III 1550 nm). For the first time, we have provided experimental evidences to show that EuB_6_ NPs can sensitize the formation of singlet O_2_ upon NIR‐II 1064 nm photoexcitation wavelength, and hydroxyl radicals by NIR‐III 1550 nm light. In vivo experiments revealed that the average half‐life of 55 d for mice treated with EuB_6_@RGD‐K NPs + NIR‐III 1550 nm light irradiation is far longer than that of EuB_6_@RGD‐K NPs + NIR‐II 1064 nm light irradiation (25 d), PBS treated mice (20 d) and EuB_6_@RGD‐K NPs‐treated mice (in dark, 18 d). Taken all together, these results suggest the successful destruction of “difficult‐to‐treat” complicated murine brain tumors using the NIR‐III combined photodynamic and photothermal therapies. To the best of our knowledge, this work is the first demonstration of destructing murine brain tumors via a multi‐functional tumor‐specific europium hexaboride‐based nanotheranostic agent to mediate MR imaging‐guided NIR‐III phototherapy. We strongly believe that the europium hexaboride‐based nanotheranostic system has a great potential to serve as an MRI contrast agent and also mediate dual functional photodynamic and photothermal therapy effects simultaneously to tackle complicated deeply seated tumors/cancers. Such unique phototheranostic systems pave a way to cross all the potential barriers involved in the translation and can be used in biomedical clinical treatments.

## 4 Experimental Section

4.1

4.1.1

##### Synthesis of EuB_6_ Nanoparticles

In a typical experiment, anhydrous europium (III) chloride and sodium borohydride (NaBH_4_) were taken in a 6:1 stoichiometric ratio and thoroughly grinded to a fine powder under argon atmosphere. The final mixture was then transferred into a quartz boat tube and placed in a tubular furnace. Prior to heating, the furnace was purged with argon gas at 100 cc min^−1^ flow rate throughout the reaction. The tubular furnace reaction was carried out at 430 **°**C for 2 h and then allowed to cool to room temperature. The as‐obtained black residue was washed with methanol and ethanol twice to remove the unreacted NaBH_4_, and further washed with HCl twice to remove the residual sodium as sodium chloride, and finally water washed to dissolve the sodium chloride. The reaction solution was centrifuged at 10 000 rpm for 12 min in which the supernatant was discarded and the pellet was dried to obtain EuB_6_ nanoparticles (EuB_6_ NPs).

##### Functionalization of RGD‐K peptide on EuB_6_ NPs

The as synthesized EuB_6_ NPs were dispersed in 10 mL of chloroform or acetonitrile solution, and to this 25 mg of F127‐COOH (Pluronic F127) polymer was added and constantly stirred for 30 min under argon atmosphere in vacuum for overnight. In the subsequent step, 5 mg of F127 polymer conjugated to EuB_6_ NPs, 3 mg of 1‐ethyl‐3‐(3‐dimethyl aminopropyl)‐carbodiimide (EDC), 2 mg of N‐hydroxy succinimide (NHS) and 1 mg of FITC labeled RGD‐K peptide (Mission Biotech) was added into this suspension and further stirred for 48 h. The final suspension was subjected for dialysis against deionized water for three times to remove the free or unconjugated RGD‐K peptide.

##### Characterization of EuB_6_ NPs

Hydrodynamic diameters and zeta potentials (*ζ*) of as‐synthesized EuB_6_ NPs were measured by dynamic light scattering (DLS), using a Zetasizer Nano instrument. UV‐vis‐NIR absorption spectra was obtained using JASCO V‐570 spectrophotometer (1 cm path length quartz cuvette) at room temperature. X‐Ray Diffraction (XRD) measurements were performed using TTrax (III) diffractometer (Rigaku, Japan). Transmission electron microscopy (TEM) images were recorded by electron microscope JEM‐2100 (JEOL, 200 KV, Japan). The phosphorescence measurements were carried out by using a luminescence spectrometer (FLS920, Edinburgh, equipped with a 450 W broad band Xenon lamp). EuB_6_ NPs (1 mg mL^−1^) were dispersed in D_2_O solution, and long pass filter (1100 nm) was introduced in‐between the sample and detector to avoid both the stray and scattering lights having wavelengths shorter than 1100 nm.

##### Quantification of RGD‐K peptide on EuB_6_ NPs

The quantification of RGD‐K peptide was performed using Bicinchonic acid assay (BCA assay). In a typical experiment, 200 μL of bicinchonic acid/copper (II) sulfate solution (50:1) was added to each well in 96‐well plate. To obtain standard curve, 50 μL solution of bovine serum albumin (BSA) was added to the sample solution and incubated at 37 **°**C for 15 min. The final solution in each well was centrifuged at 10 000 rpm, and the supernatant was collected to measure their respective optical densities using an enzyme linked‐immunosorbent assay (ELISA) plate reader at 562 nm.

##### Photostability of EuB_6_ NPs

In a typical experiment, 1 mg mL^−1^ of EuB_6_ NPs suspension was taken into a glass vial and then irradiated with 1550 nm CW laser (500 mW cm^−2^) for 30 min. The changes in the absorption spectra as a function of time was monitored using UV‐vis‐NIR absorption spectrophotometer. In addition, the morphology of particles before and after photoirradiation was also monitored using TEM.

##### Photothermal Conversion Efficiency of EuB_6_ NPs

The photothermal conversion efficiency of EuB_6_ NPs was calculated by using the previously reported literature procedures^[^ ^43^ ^]^

(1) $$\eta =\;\frac{hs\left(T_{\text{max}}-\;T_{\text{surr}}\right)-\;Q_{\text{dis}}}{I\left(1-10^{-\text{A1550}}\right)}$$

where ‘*η*’ is the photothermal conversion efficiency, ‘*h*’ is heat transfer coefficient and ‘*s*’ is the cuvette surface area. The maximum temperature of the EuB_6_ NPs solution is 49.6 **°**C and surrounding temperature is 20 **°**C. The laser output power intensity (*I*) is 300 mW cm^−2^, the absorbance of EuB_6_ NPs is 0.5 and *Q*_dis_ indicates the dissipated heat from the light absorbed by cuvette and solvent.

In order to calculate *hs*, a dimensionless parameter is introduced ‘*θ*' as follows

(2) $$\theta \;=\;\frac{T\;-T_{\text{surr}}}{T_{\max}\;-T_{\text{surr}}}$$

According to Figure S9, Supporting Information, *τ*_s_ was determined and calculated to be 206 s

(3) $$hs\;=\;\frac{m_{D}\;-C_{D}}{\tau_{s}}$$

where *m* is 0.2 g and C is 4.2 J g^−1^ **°**C. Therefore, according to equation (3), the value of *hs* is 4.07 mW **°**C^−1^. *Q*_dis_ is heat dissipated from sample solution and it was measured to be 40 mW. By substituting all the obtained values in the above equation (1), the photothermal conversion efficiency of the EuB_6_ NPs can be calculated to be 39.2%.

##### EPR Measurements

Electron paramagnetic resonance (EPR) experiments were performed on a Bruker EPR spectrometer at room temperature. For the detection of singlet oxygen (^1^O_2_) radicals using electron paramagnetic resonance (EPR), 500 μL of 1 mg mL^−1^ and EuB_6_ NPs aqueous suspension was added to 40 μL of 1.0 M DMPO in H_2_O. The resulting solution was thoroughly mixed and then subjected to 1064 and 1550 nm laser irradiations with a power intensity of 1 W cm^−2^ right in front of the EPR spectrometer during the measurements. Instrument parameter settings: scan width, 100 G; time constant, 40.96 ms; microwave power, 10.12 mW; frequency, 9.8 GHz; time constant, 40.96 ms; scan width, 100 G.

##### Cell Culture

ALTS1C1 cells (murine astrocytoma) were obtained from Bioresource Collection and Research Center (BCRC, Taiwan). The cells were grown in Dulbecco's modified Eagle's medium (DMEM, Invitrogen) with 10% heat‐inactivated fetal bovine serum (Invitrogen, Carlsbad, CA, USA), 100 μg mL^−1^ penicillin and 100 U mL^−1^ streptomycin. The cells were grown in humidified incubator at 37 °C (95% humidity, 5% CO_2_).

##### Cellular Uptake

The cellular uptake of EuB_6_@RGD‐K NPs in ALTS1C1 cells were analyzed by using confocal laser scanning optical microscope. ALTS1C1 cells were seeded at a density of 3 × 10^5^ cells mL^−1^ on a 6‐well plate by placing cover slips and incubated overnight. The cells were then treated with different concentrations of EuB_6_@RGD‐K NPs to each well and incubated for another 12 h. The cells were washed with a phosphate buffered saline (pH 7.4) twice and further fixed with P‐formaldehyde (4%) solution followed by treated with PBST (5% Tween‐20 in PBS) solution three times. The ALTS1C1 cells were stained with DAPI dye and samples were examined under a confocal laser scanning microscopy (Zeiss, LSM 700).

##### In Vitro Molecular Targeting Using EuB_6_@RGD‐K NPs

ALTS1C1 cells (2 × 10^4^ cells mL^−1^) were cultured in a 6‐well plate by placing a cover slip in each well, and incubated overnight. The cells were then feeded with various concentrations of EuB_6_ NPs (without RGD peptide) and FITC‐labeled EuB_6_@RGD‐K NPs and incubated for 12 h. The cells were then washed with phosphate buffered saline (PBS) followed by trypsinization and centrifuged at 10 000 rpm for 5 min. Discard the supernatant and then resuspended in PBS and analyzed using flow cytometry under FITC channel.

##### In Vitro Phototoxicity Experiments

200 μL of ALTS1C1 cells containing solution (2 × 10^4^ cells mL^−1^) was added to each well of a 96‐well plate and incubated for overnight. Aliquots of aqueous solution containing different concentrations of EuB_6_@RGD‐K NPs were added to the 96‐well plate and incubated for 4 h. The phototoxicities were then estimated by irradiating EuB_6_@RGD‐K NPs internalized ALTS1C1 cells with 1064 nm (300 mW cm^−2^; 10 min) and 1550 nm (300 mW cm^−2^; 7 min). The irradiation times were adjusted in such a way that the total number of photons being absorbed by the EuB_6_@RGD‐K NPs at both wavelengths to be the same by taking into account the relative magnitudes of absorbances at 1064 and 1550 nm respectively. After irradiation, the ALTS1C1 cells were further incubated at 37 °C for another 12 h. A 10 μL amount of a MTT (3‐(4,5‐dimethylthiazol‐2‐yl)‐2,5‐diphenyl tetrazolium bromide) aqueous solution (0.5 mg mL^−1^) was added to each well and the tumor cells were further incubated for another 4 h. Then, the supernatant in the 96‐well plate was discarded, and then add 1 mL of DMSO into each well to lyse out the cell membrane, followed by stirring. Then the final solution in each well was centrifuged at 10 000 rpm for 5 min, and the upper solution was collected to measure their respective optical densities using an enzyme linked‐immunosorbent assay (ELISA) plate reader at 570 nm. Based on the calibration curve, absorbance values were then converted into cellular viabilities.

##### LIVE/DEAD Assay Using Calcein AM and PI Staining

ALTS1C1 cells (2 × 10^4^ cells mL^−1^) were cultured in a 6‐well plate by placing the cover slip inside each well. After 24 h of incubation, various concentrations of EuB_6_@RGD‐K NPs were added to each well and incubated for 12 h at 37 °C. The cells were then washed with PBS and subjected to photoirradiation using 1064 and 1550 nm lasers, respectively. After photoirradiation, the cells were then kept inside the incubator overnight. The cells were then stained with 10 μg mL^−1^ of Calcein AM and 50 μg mL^−1^ of propidium iodide (PI) for 20 min and then examined using fluorescence microscope.

##### Heat Shock Protein (HSP 70) Studies

ALTS1C1 cells (2 × 10^6^ cells/well) were seeded into 12‐well plates and incubated overnight. Then different amounts of EuB_6_@RGD‐K NPs were added to the cells and incubated for 4 h. The cells were then washed with PBS and subjected to photoirradiation using 1064 and 1550 nm CW lasers, respectively, followed by staining with the Alexa Fluor 640 conjugated HSP 70 antibody (1:50 dilution; Cell signaling, USA). Cells were then trypsinized, washed and finally resuspended in 1 mL PBS buffer, followed by flow cytometry analysis.

##### In Vivo Fluorescence Imaging

All the mice (C57BL/6J male; 20–25 g; 7–8 weeks old) used in this study were purchased from Animal Central Core Facility (National Taiwan University, Taipei, Taiwan). The animal experiments were performed with the approval of National Tsing Hua University Animal Center (Institutional Animal Care and Use Committee, IACUC approval number 10 543). A brain tumor model was established by implanting 2 μL of ALTS1C1 cells (at a density of 2 × 10^7^ cells mL^−1^ in F‐12 K medium) injected into the mice through the intracranial injection. The tumor implantation was performed under anesthetic conditions using Zoletil‐Rompun (4:1 ratio) through intraperitoneal injection. For in vivo fluorescence imaging experiments, ALTS1C1 tumor bearing mice were intravenously injected with Cy5.5‐encapsulated EuB_6_@RGD‐K NPs (150 μg mL^−1^, 60 μ L^−1^/mouse) or PBS (100 μL/mouse). After 14 h post intravenous injection, fluorescence imaging was performed using IVIS imaging system under anesthetic conditions. NIR fluorescence images were acquired using an IVIS Xenogen (Cy5.5 channel, *λ*_ex_ = 615–665 nm, *λ*_em_ = 695–770 nm).

##### In Vivo Monitoring Tumor Temperature

After being intravenously injected with PBS and EuB_6_@RGD‐K NPs into tumor bearing mice, each group of mice were photoirradiated with either 1064 or 1550 nm light irradiation. The temperature was monitored at the tumor sites using a NIR thermal camera (Thermo shot F30) and the images were analyzed using TAS 19 software.

##### In Vivo Biodistribution of EuB_6_@RGD‐K NPs by ICPMS

ALTS1C1 tumor bearing mice were injected with EuB_6_@RGD‐K NPs (50 mg kg^−1^) via intravenous injection to the brain. In order to disrupt the blood brain barrier (BBB) temporarily in mice, mannitol was injected intravenously 30 min before injection of EuB_6_@RGD‐K NPs. Mannitol is known to be able to cause short time reversible shrinkage of endocellial cells, and thus allows opening the blood brain barrier (BBB) for 30–60 min.^[^ ^44^, ^45^ ^]^ After different time points of post intravenous injection (8, 12, and 24 h), mice were sacrificed and organs such as liver, kidney, spleen, blood, and tumor were harvested. The mice organs were digested using hydrogen peroxide (H_2_O_2_, 40%) and perchloric acid (HClO_4_, 60%) and followed by the addition of hydrofluoric acid in (1:2 vol%). The organs were then incubated at 50 °C followed by ultrasonication for 1 d.

##### In Vivo Tumor Growth Study

For in vivo studies, C57BL/6J mice (4 to 6 weeks old) were purchased from the Animal Central Core Facility at National Applied Research Laboratories, Taiwan. The protocols for animal use were approved by the Institutional Animal Care and Use Committee (IACUC) of National Tsing Hua University, Taiwan. To perform, in vivo tumor growth study, ALTS1C1 tumor bearing mice were randomly divided into 4 groups at day 0 (each group of mice, *n* = 6). Out of four groups, one group of mice were injected with PBS and another group of mice were injected with EuB_6_@RGD‐K NPs (50 mg kg^−1^) without any photoirradiation. The remaining two groups of mice were intravenously injected with EuB_6_@RGD‐K NPs and subjected to photoirradiation using 1064 nm laser (300 mW cm^−2^; 10 min) and 1550 nm laser (300 mW cm^−2^; 7 min), respectively. The body weights of the mice were recorded every day and the tumor volumes were measured using *T*_1_‐weighted MR imaging. *T*_1_‐weighted in vivo MR images were recorded for brain and whole body. All *T*_1_ weighted MR images were recorded using 7 T MR imaging system (Biospec 70/30 USR; Bruker at National Taiwan University, Taiwan) through coronal cross‐sectional view. The following parameters were used for all *T*_1_ weighted MR images recorded: TR = 800 ms; TE = 9 ms; FA = 180; matrix = 256 × 256; FOV = 20 × 20 mm^2^.

##### Immuno‐Histochemical (IHC) Analysis

For histopathology studies, one of the mice from each group was sacrificed, and the major organs (such as tumor, liver, and spleen) were harvested, and fixed in 10% formalin solution. The tissues were embedded in paraffin, and sectioned at 10 μm thickness followed by staining with hematoxylin and eosin (H&E). Immunohistological examination was used to study the extent of tissue damage before and after the phototherapy treatments.

##### Serum Analysis

To assess the hepatic and renal functioning for the mice injected with EuB_6_@RGD‐K NPs, serum levels were measured in the treated mice group and compared to that of the control mice. This included three assays of hepatic function (Alanine Aminotransferase (ALT), Aspartate Aminotransferase (AST) and Alkaline Phosphatase (ALP)), and two renal assays (Urea nitrogen and creatinine) performed by a blood biochemical autoanalyzer.

##### Light Penetration Experiments

The percentage of light penetration through mouse skulls was determined using 1064 and 1550 nm laser sources at various power densities of 0.2, 0.3, 0.4, and 0.5 W m^−^^2^. Light penetration through pork tissue was measured by placing pork tissue samples of 1, 2, 5, 7.5, or 10 mm thickness in between a laser power density of 0.5 W cm^−^^2^ and a power detector. The percentage of light penetration was defined as the fraction of light detected after the mouse skull or a pork tissue, relative to the light intensity output from the light source.

##### Measurements of ROS inside Cancer Cells

Intracellular ROS generation was measured using the fluorescent probe 2,7‐dichlorofluorescein diacetate (DCFH‐DA). ALTS1C1 cells were cultured in 6‐well plates at a density of 2 × 10^5^ cells mL^−1^. EuB_6_@RGD‐K NPs solutions were then added, and the cells were incubated for 12 h. Subsequently, the cells were treated with 5 μM DCFH‐DA solution and incubated for 30 min. Green fluorescence was measured by flow cytometry using the FITC channel. The cells were then photo‐irradiated with 1064 nm (300 mW cm^−^^2^ for 10 min), and 1550 nm (300 mW cm^−^^2^ for 7 min**)** light under identical conditions.

##### Measurements of Singlet Oxygen Levels inside Cancer Cells

ALTS1C1 cells were cultured in a 6‐well plate at a density of 2 × 10^5^ cells per well. After 24 h of incubation, EuB_6_@RGD‐K NPs solution was added and incubated for an additional 12 h. Following this, 1 mM SOSG (Invitrogen, USA) was added to the cell solution and incubated for 30 min. The cells were then photo‐irradiated with 1064 nm (300 mW cm^−^^2^ for 10 min), and 1550 nm (300 mW cm^−^^2^ for 7 min) light and were analyzed by flow cytometry.

## Conflict of Interest

The authors declare no conflict of interest.

## Supporting information

Supplementary Material

## Data Availability

The data that support the findings of this study are available in the supplementary material of this article.
