# Supplementary material for: Engineering Tumor‐Specific Nanotheranostic Agent with MR Image‐Guided NIR‐II & ‐III Photodynamic Therapy to Combat Against Deeply Seated Orthotopic Glioblastoma
Source: Small Sci. 2024 Jul 14;4(10):2400191. doi: 10.1002/smsc.202400191 (PMC11935268; doi:10.1002/smsc.202400191)
Supplement: Supplementary file 1 — Supplementary Material [file SMSC-4-2400191-s001.pdf]

**Supplementary Information**

**Engineering Tumor-Specific Nanotheranostic Agent with MR Image-Guided NIR II & III Phototherapy to Combat Against Deeply Seated Orthotopic Glioblastoma**

Karthik Nuthalapati[1], Raviraj Vankayala[2]*, Munusamy Shanmugam[1], Suresh Thangudu[1], Chi-Shiun Chiang[3], Kuo Chu Hwang[1]*

[1][*] Prof. Kuo Chu Hwang, Dr. Karthik Nuthalapati, Dr. Munusamy Shanmugam, Dr. Suresh Thangudu
Department of Chemistry, National Tsing Hua University, Hsinchu 30013, Taiwan ROC. *E-mail: kchwang@mx.nthu.edu.tw.

[2][*] Dr. Raviraj Vankayala,
Department of Bioscience and Bioengineering, Interdisciplinary Research Platform Smart Healthcare, Indian Institute of Technology Jodhpur, Karwar 342030, India. *Email: rvankayala@iitj.ac.in.

[3]Prof. Chi-Shiun Chiang,
Department of Biomedical Engineering and Environmental Sciences, National Tsing Hua University, Hsinchu 30013, Taiwan ROC.

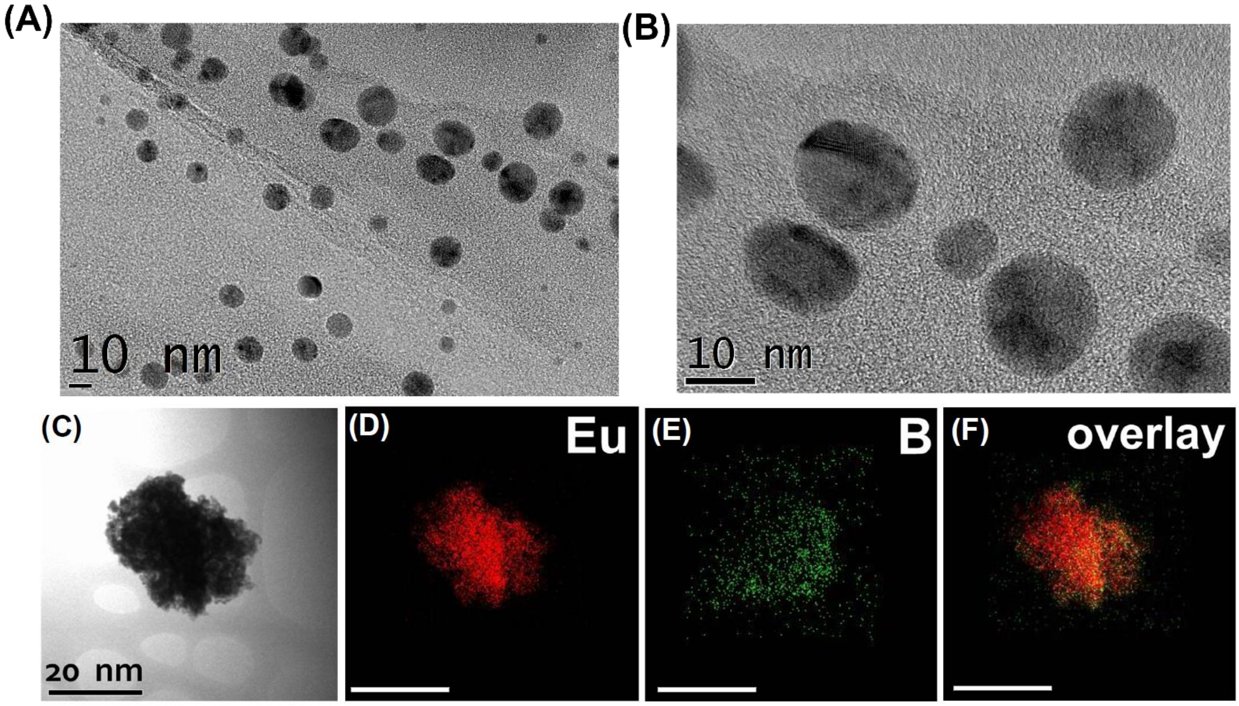


**Figure S1.** (A) and (B) represents the TEM images of as synthesized $EuB_6$ NPs. (C) STEM image and (D), (E) and (F) elemental mapping image of $EuB_6$@RGD-K NPs. Eu and B are shown as red and green color, respectively.

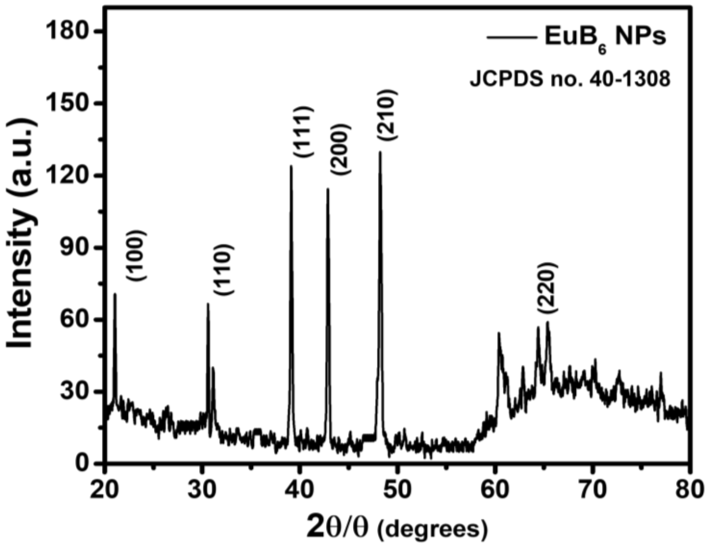


**Figure S2.** Powder X-ray diffraction (PXRD) spectrum of $EuB_6$ NPs.

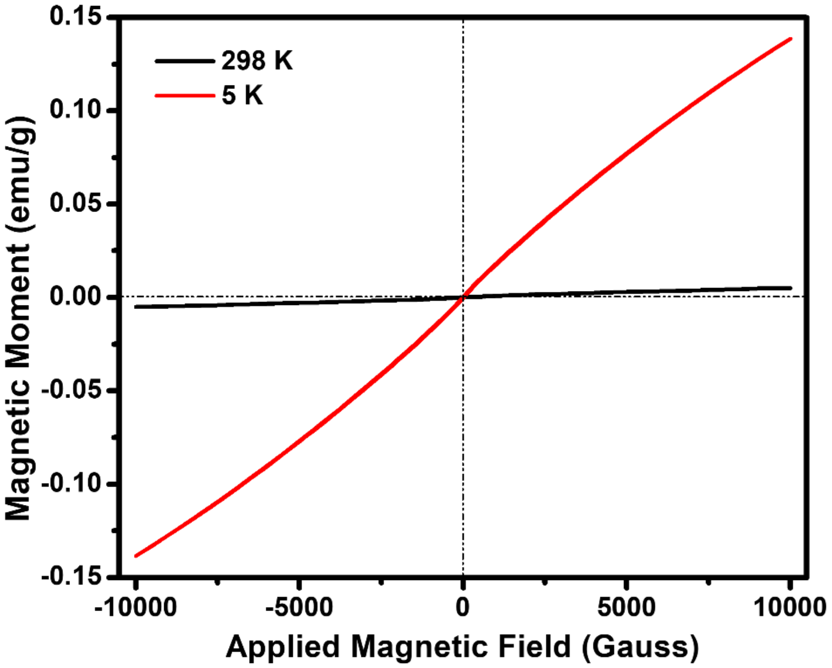


**Figure S3.** SQUID analysis for the as synthesized $EuB_6$ NPs at both 5K and 298K temperatures.

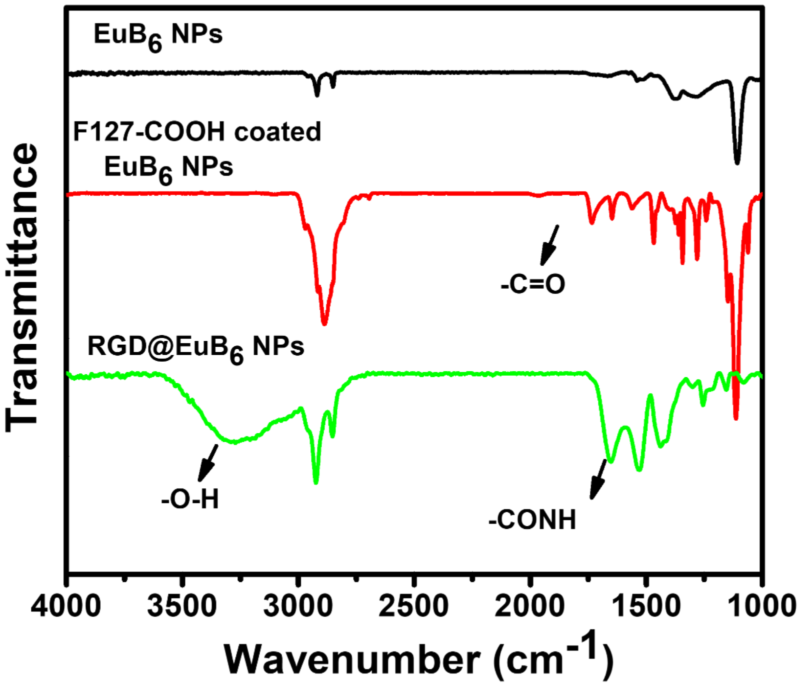

**Figure S4.** Fourier Transform Infrared (FTIR) spectra for $EuB_6$ NPs, F127-COOH coated $EuB_6$ NPs and RGD@$EuB_6$ NPs, respectively.

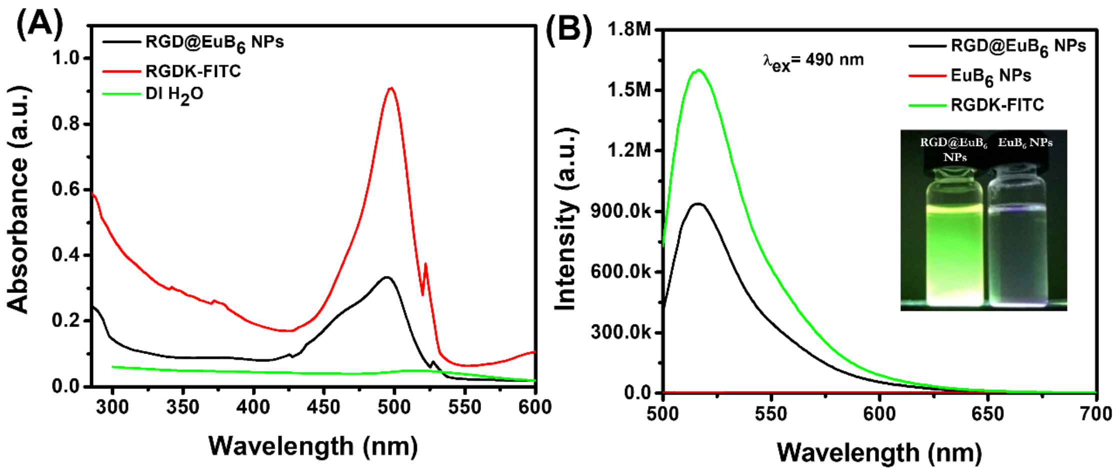


**Figure S5.** (A) UV-vis absorption and (B) Fluorescence emission spectra for $EuB_6$ NPs, RGDK-FITC and RGD@$EuB_6$ NPs, respectively.

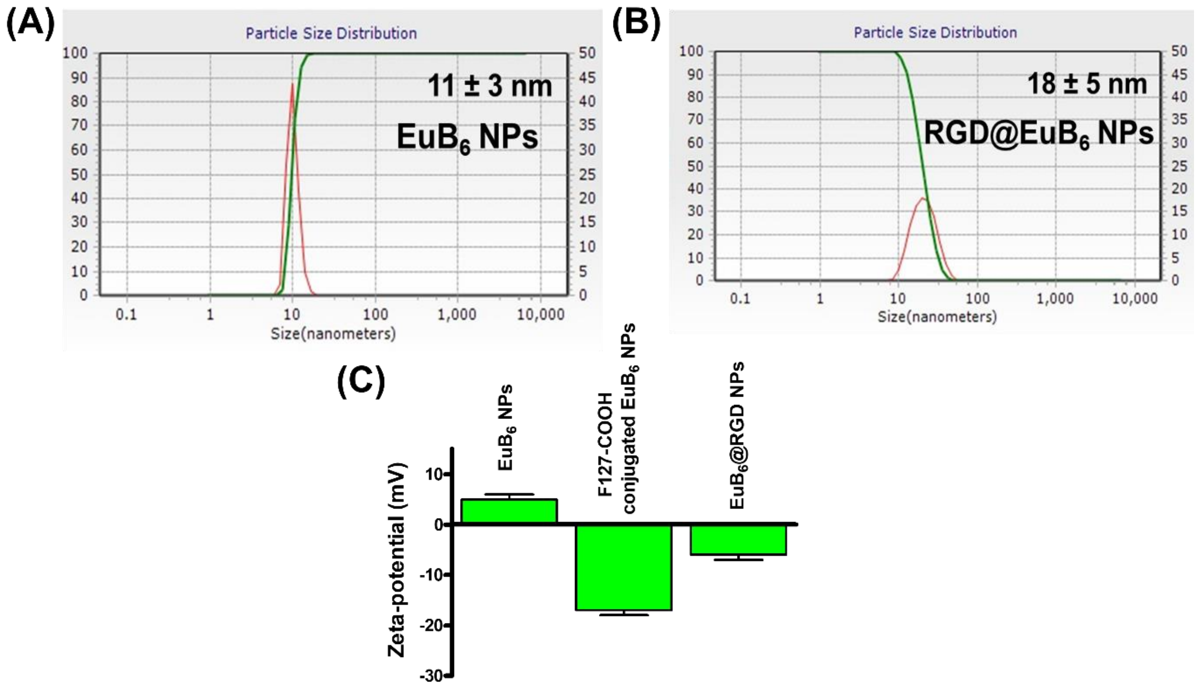


**Figure S6.** (A) and (B) represents the dynamic light scattering (DLS) spectra and (C) Zeta-potential for $EuB_6$ NPs and RGD@$EuB_6$ NPs.

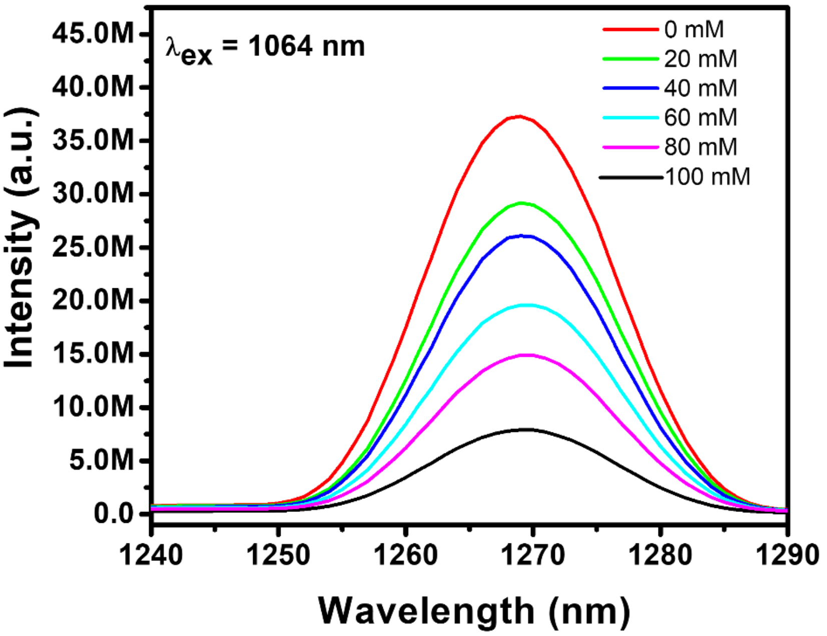


**Figure S7.** Singlet $O_2$ phosphorescence emission spectra for $EuB_6$ NPs in the presence of different concentrations of sodium azide.

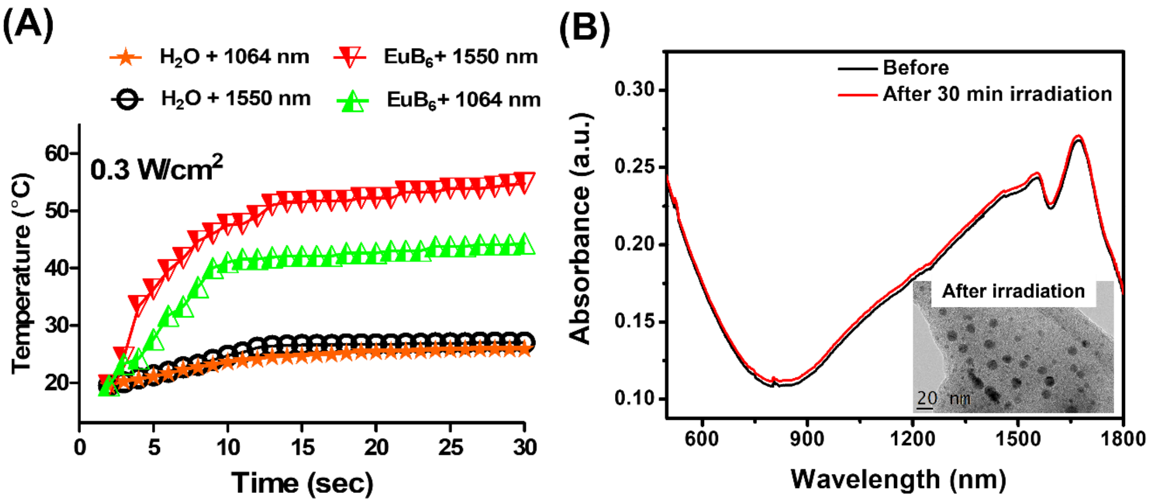


**Figure S8.** (A) Photothermal temperature rise profiles for $EuB_6$ NPs using NIR-II 1064 nm and NIR-III 1550 nm light irradiation conditions. (B) UV-vis-NIR absorption spectra for $EuB_6$ NPs before and after 30 min of NIR-III 1550 nm light irradiation conditions. The inset represents the TEM image of $EuB_6$ NPs after photoirradiation.

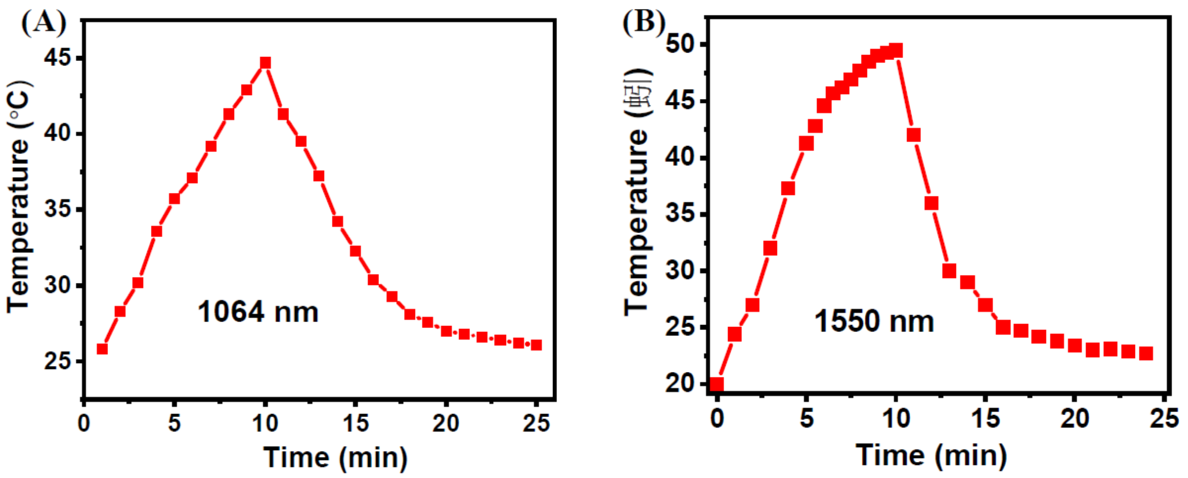


**Figure S9.** Photothermal conversion efficiencies of $EuB_6$@RGD-K NPs upon (A) NIR-II 1064 nm, and (B) NIR-III 1550 nm NIR light irradiation, respectively

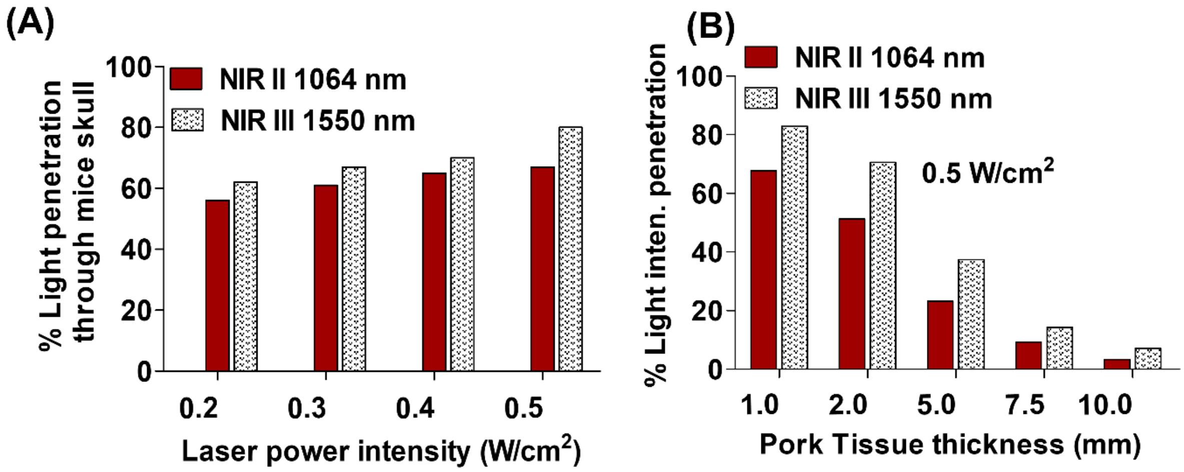


**Figure S10.** (A) Percentage of light penetration through the mice skull, and (B) percentage of light intensity penetration as a function of pork tissue thickness in millimeters using NIR-II 1064 nm and NIR-III 1550 nm light, respectively. The statistically significant differences are indicated as $*p < 0.05$, $**p < 0.01$, and $***p < 0.001$.

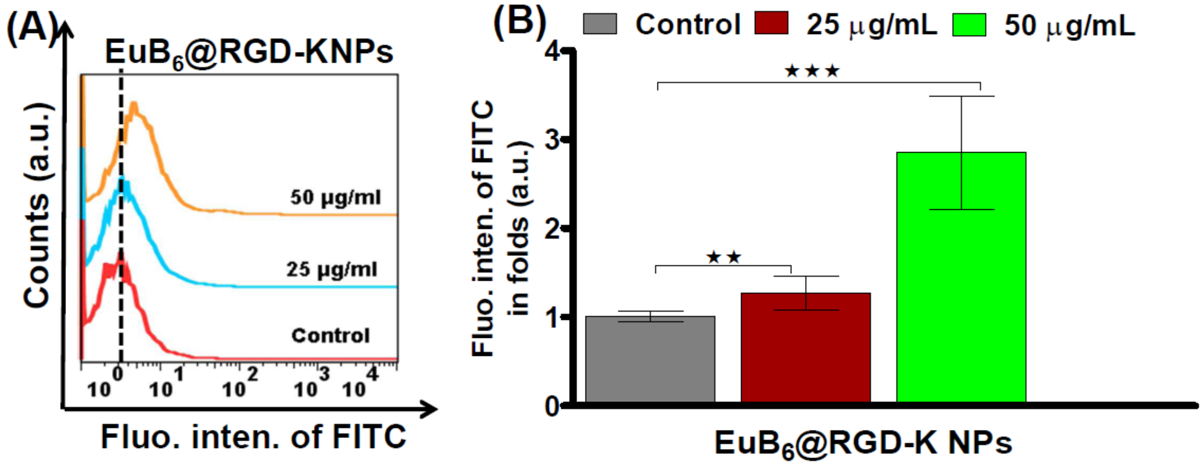


**Figure S11.** Cellular uptake of $EuB_6$@RGD-K NPs by ALTS1C1 cancer cells was monitored by measurements of FITC green fluorescence using (A) flow cytometry, and (B) the relative fluorescence intensity as a function of the amounts of $EuB_6$@RGD-K NPs fed in the solution. The RGD-K peptide was labeled with a FITC green fluorescence dye (product ID:990843, Mission Biotech). The statistically significant differences are indicated as $*p < 0.05$, $**p < 0.01$, and $***p < 0.001$.

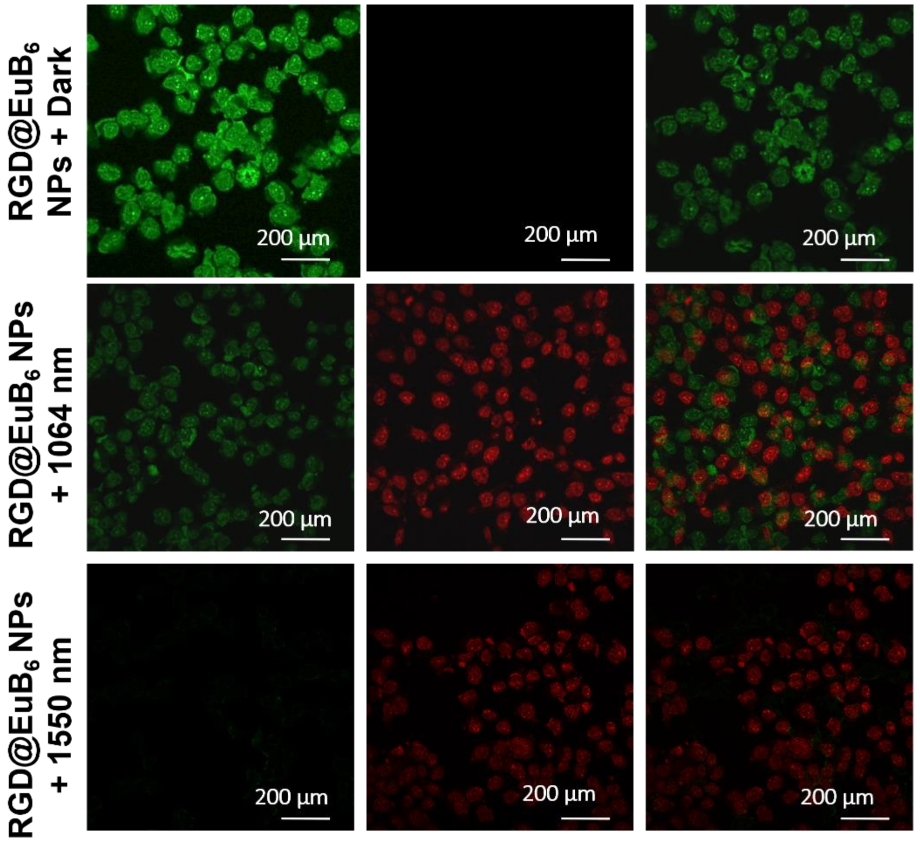


**Figure S12.** LIVE/DEAD assay monitored using fluorescence microscopy for RGD@$EuB_6$ NPs in dark and light irradiation conditions. The green and fluorescence were measured using FITC and PI channels.

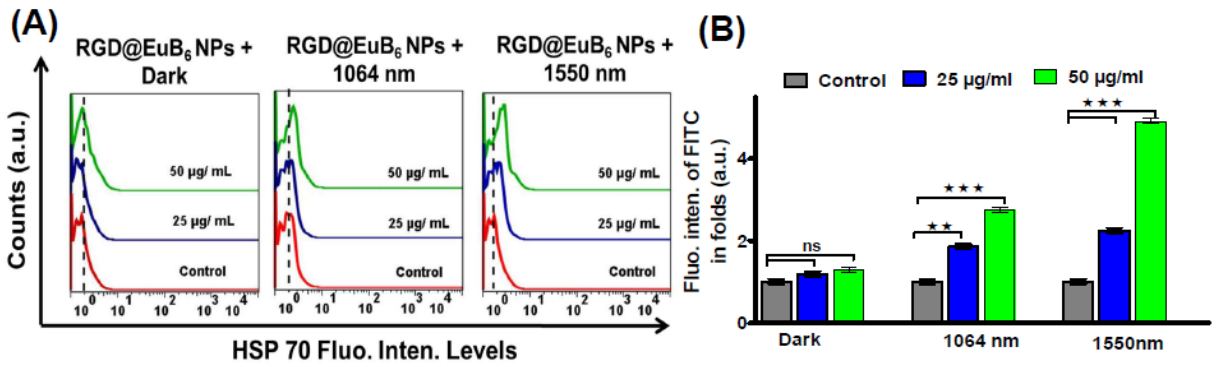


**Figure S13.** Heat shock protein expression (HSP70) levels of RGD@EuB$_6$ NPs-internalized ALTS1C1 cells under dark and light irradiation conditions monitored using flow cytometry. The red fluorescence is measured using PI channel.

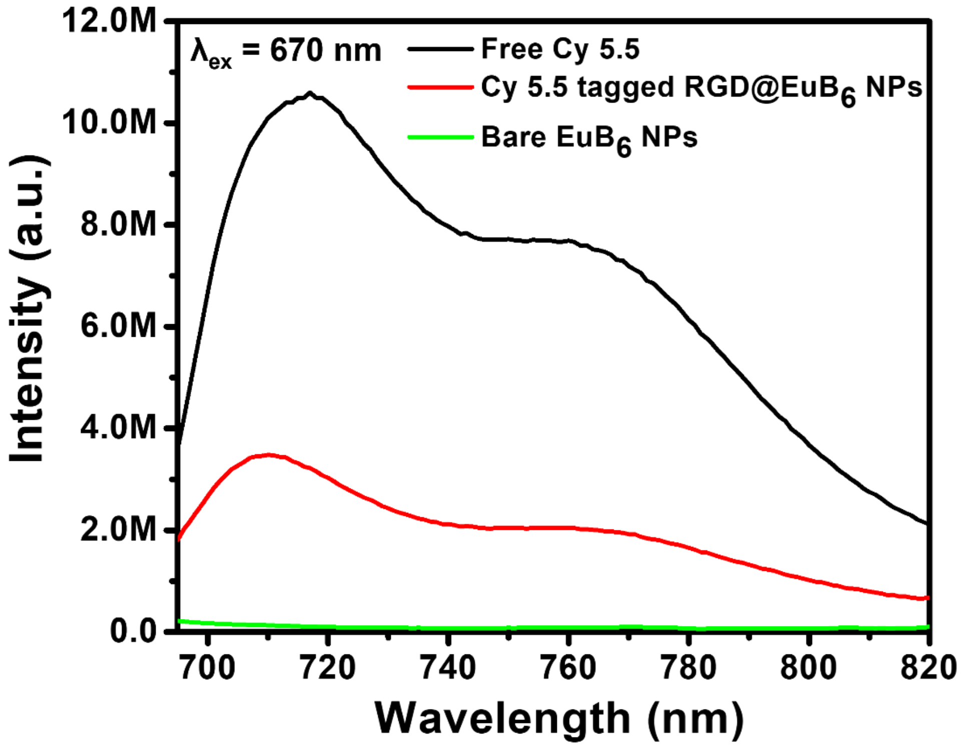


**Figure S14.** Photoluminescence emission spectra of Cy5.5 tagged RGD@EuB$_6$ NPs

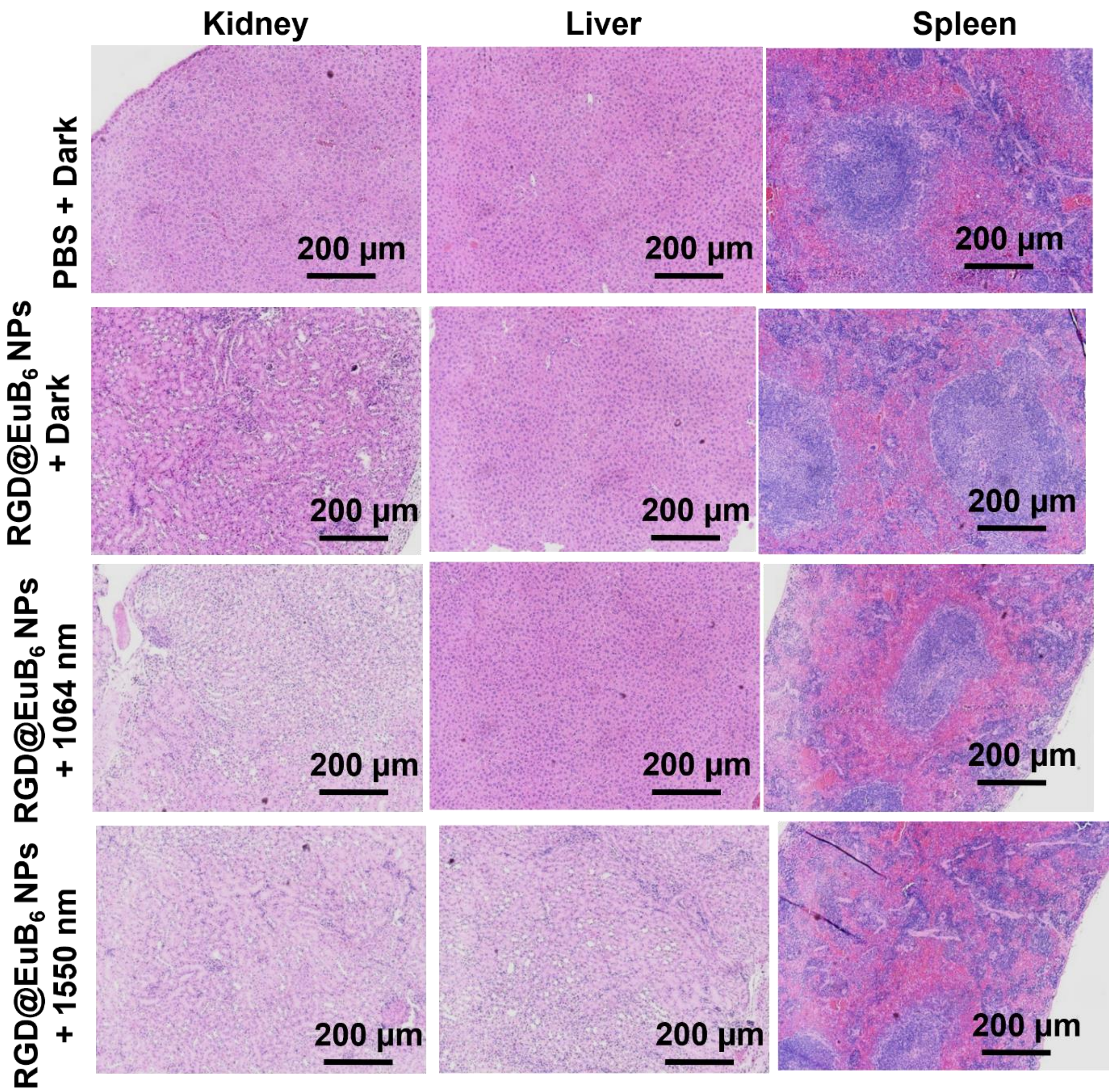


**Figure S15.** Histopathological examination of major organs such as liver, kidney and spleen for the mice injected with PBS and RGD@$EuB_6$ NPs in dark and exposed with NIR-II 1064 nm and NIR-III 1550 nm light irradiations.

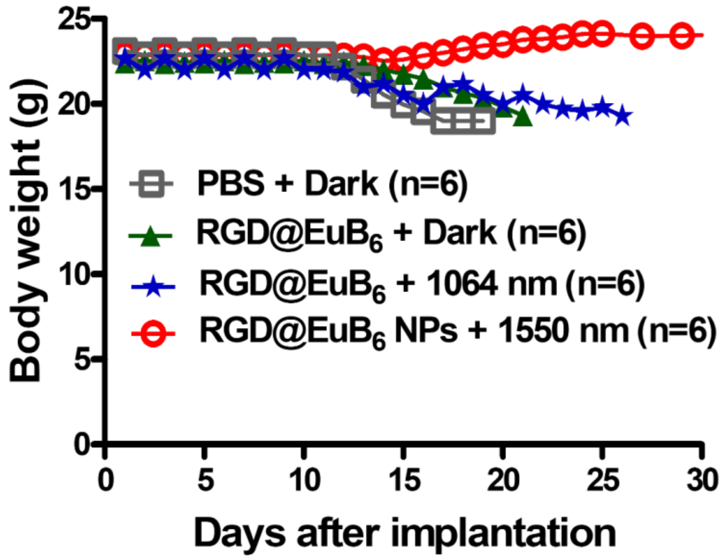


**Figure S16.** Body weights of the mice for various groups monitored during the treatment period.

| Parameter | Control mice | Treated mice | Range |
|---|---|---|---|
| AST (units/l) | 56 | 65 | 5-60 |
| ALT (units/l) | 83 | 76 | 5-100 |
| ALP (units/l) | 50 | 55 | 20-150 |
| BUN (mg/dl) | 25 | 22 | 6-30 |
| CRE (mg/dl) | 0.1 | 0.4 | 0.3-1.5 |

**Abbreviations:** AST (Aspartate Transferase); ALT (Alanine Transferase); ALP (Alkaline Phosphatase); BUN (Blood Urea Nitrogen); CRE (Creatinine)

**Figure S17.** Assessment of liver and kidney functions for the mice injected with both PBS- and RGD@$EuB_6$ NPs treated mice at 24 hours post intravenous injection.

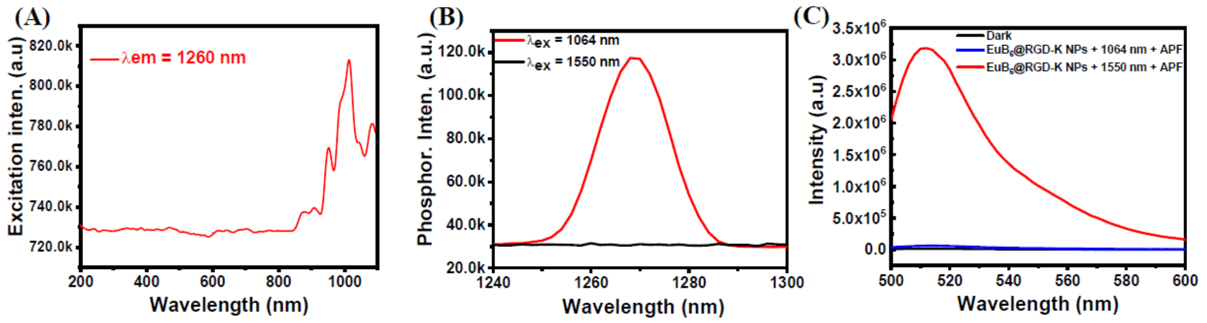


Figure S18. (A) Excitation spectra of $EuB_6$@RGD-K NPs for singlet oxygen phosphorescence emission @1260 nm. (B) singlet oxygen phosphorescence emission spectra from $EuB_6$@RGD-K NPs upon 1064 and 1550 nm NIR light irradiation, respectively. (C) APF emission spectra from $EuB_6$@RGD-K NPs upon 1064 and 1550 nm NIR light irradiation, respectively.

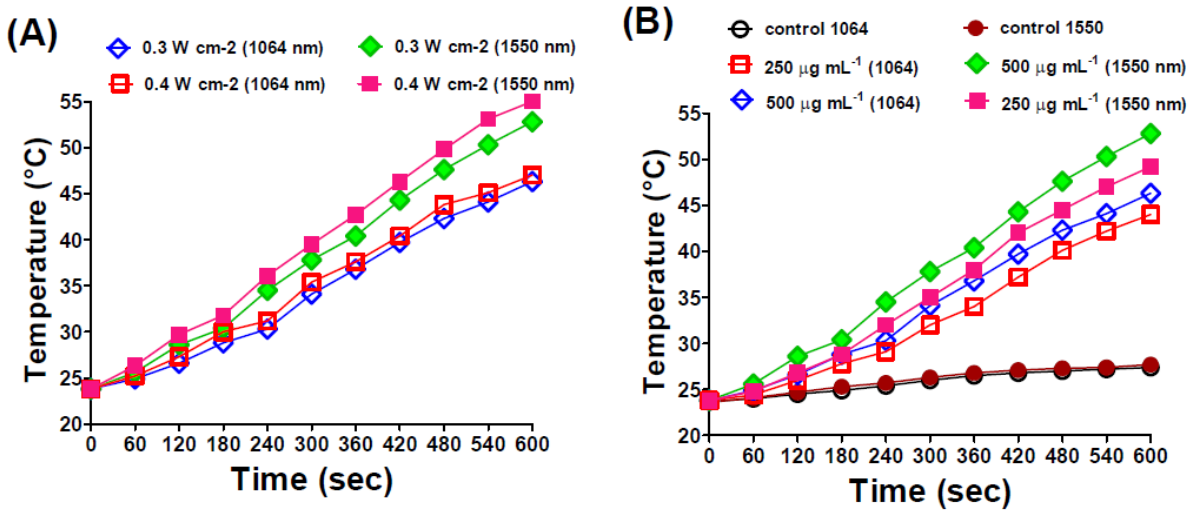


**Figure S19**. Photothermal performance of $EuB_6$@RGD-K NPs under 1064 and 1550 nm NIR light irradiation with (A) different laser powers, and (B) different NPs concentrations, respectively.

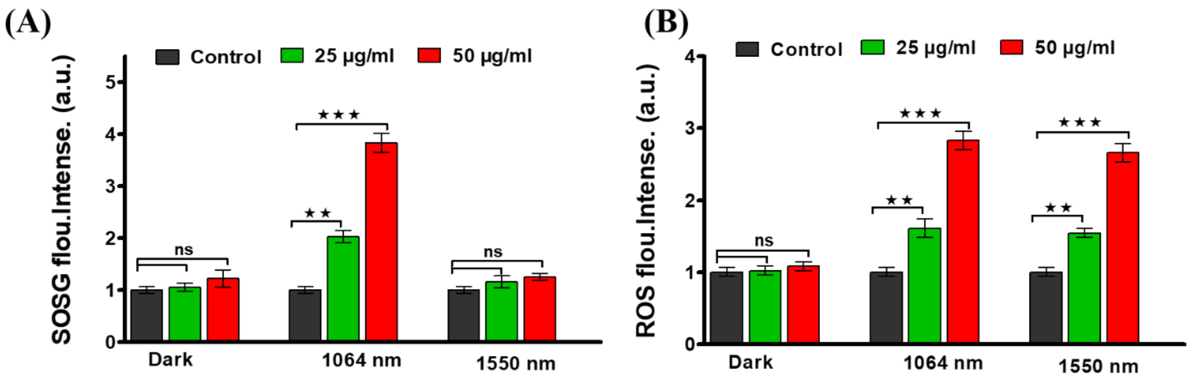


**Figure S20** (A) SOSG fluorescence levels (corresponding the singlet oxygen levels) of $EuB_6$@RGD-K NPs-internalized ALTS1C1 cancer cells, and (B) ROS generation monitored

by DCFH-DA fluorescence using flow cytometry at different concentrations under dark, 1064 or 1550 nm light irradiation, respectively. The statistically significant differences are indicated as $*p < 0.05$, $**p < 0.01$, and $***p < 0.001$.

***Quantum yield measurement of singlet oxygen from $EuB_6$ NPs***

To determine the quantum yield of singlet $O_2$ formed via sensitization by $EuB_6$ NPs, we use methylene blue as a reference, and determine the singlet $O_2$ formation yield of $EuB_6$ NPs by comparing the phosphorescence emission area of singlet $O_2$ (integration area from 1225 to 1300 nm) with that obtained using methylene blue as a photosensitizer.

Phosphorescence emission area of singlet $O_2$ sensitized by methylene blue, $Area_{phos}$, MB, is equal to the multiplication product between the absorbance of methylene blue at 650 nm ($Ab_{MB-650}$) and the singlet $O_2$ sensitization quantum yield ($\Phi_{MB}$), i.e., the equation (1).

The incident light intensity at 650 nm is 2 mW/cm$^2$ (slit width: 10 nm) from the luminescence spectrometer. The quantum yield for singlet $O_2$ formation by MB in $D_2O$ is 0.68.[S1]

$Area_{phos\text{-}MB} = Ab_{MB\text{-}510} \times I_{650} \times \Phi_{MB}$ …………… (1)
$Area_{phos\text{-}MB} = 716907$
Similar equation can be derived for singlet $O_2$ phosphorescence as sensitized by $EuB_6$ NPs (see equation (2))
$Area_{phos\text{-}EuB6} = Ab_{EuB6\text{-}1064} \times I_{1064} \times \Phi_{EuB6}$ ……………...(2)
$Area_{phos\text{-}EuB6} = 674376$
The incident light intensity of FLS 920 spectrometer at 1064 nm is 7 mW/cm$^2$ (slit width: 10 nm). Both, methylene blue and $EuB_6$ NPs solutions absorbance was adjusted to be 0.5 at their respective excitation wavelengths. After substituting all the parameters and dividing equations (1)/(2), one can obtain the value of $\Phi_{EuB6}$ is 0.187.

***Measurements of photothermal conversion efficiencies from $EuB_6$ NPs upon 1064 nm and 1550 nm NIR light irradiation***.

The photothermal conversion efficiency (PCE) of $EuB_6$@RGD-K NPs was evaluated following the literature procedure.[S2] PCE value of $EuB_6$@RGD-K NPs upon 1064 and 1550 nm light irradiation was estimated using the following equations. PCE can be calculated using the expression

$$\eta = [hS\,(T_{max} - T_{surr}) - Q_{dis}] / I_0\,(1\text{-}10^{-A_{808}}) \;………………..… (1)$$

The laser power ($I_0$) is 300 mW/cm$^2$, the absorbance of $EuB_6$@RGD-K NPs at 1064 nm is 0.5. Where $h$ represents the heat transfer coefficient, $S$ is the surface area, $T_{max}$ is the maximum temperature, and $T_{surr}$ is the surrounding temperature. The value of $hS$ was estimated using the following expression

$hS = mD \times cD / \zeta s$ ………………………………………. (2)

Heat dissipation time constant ($\zeta s$) was determined by plotting the linear data of cooling period with the negative natural logarithm as expressed below

$t = - \zeta s \ln(\theta)$……………………………………………..…(3)

$\theta = [T - T_{surr}]/ [T_{max} – T_{surr}]$……………………………….(4)

$T_{surr}$ represents the surrounding temperature 25.8 °C and $T_{max}$ is the maximum temperature of 44.9 °C (**see** Figure S9 above**).** Upon substituting in equation 2,

$hS = 0.4*4.6/469$ J/sec $^{o}$C = 3.92 mW/ $^{o}$C

$\eta_{1064\ nm} = 3.92*(19.9) -12.83/ 300 (1-10^{\wedge -0.5})$ = **31.4 %**

The photothermal conversion efficiency of $EuB_6$@RGD-K NPs at 1550 nm ($\eta_{1550\ nm}$) can be calculate in the similar way. The laser power ($I_0$) is 300 mW/cm$^2$, the absorbance of $EuB_6$@RGD-K NPs at 1550 is 0.5 and $T_{max} – T_{surr}$ is 26.8. On substituting in equation (1) we obtain $\boldsymbol{\eta_{1550\ nm}}$ **is 39.2%.**

***Quantum yield measurements for the generation of hydroxyl radicals from*** $EuB_6$@RGD-K NPs.

1 mg/mL of $EuB_6$@RGD-K NPs and 10 μL of APF (aminophenyl fluorescein, a ROS fluorescence sensor, from Lifespan Biosciences, USA) were mixed in $H_2O$ and irradiated with 1550 nm CW lasers at 300 mW/cm² for 5 min. The resulting supernatant solution was then subjected to fluorescence emission measurement in the range of 500-600 nm, using 490 nm light for photoexcitation. Both the excitation and emission slit widths were set to be 3 nm. The hydroxyl radical generation quantum yield of $EuB_6$@RGD-K NPs under 1550 nm irradiation was measured using $TiO_2$ NPs as reference standard.[S3] The quantum yield for the formation of hydroxyl radicals from $TiO_2$ NPs was reported to be 0.04 upon 375 nm light irradiation. APF was used as both a •OH radical trapping reagent and a fluorescence probe for both systems. The light intensity of the Hg lamp was 420 mW/cm² at 375 nm, and 300 mW/cm² at 1550 nm. The fluorescence area ($Area_{fluo-TiO2+APF}$) in the range of 500-600 nm was measured for $TiO_2$ NPs.

$Area_{fluo-TiO2+APF} = Ab_{TiO2-375\ nm} \times I_{375\ nm} \times \Phi_{TiO2-OH}$ …………… (1)

$Area_{fluo-TiO2+APF} = 2034349$

Similar equation can be derived for $EuB_6$@RGD-K NPs NPs.

$Area_{fluo-EuB6@RGD-K\ NPs-1550+APF} = Ab_{EuB6@RGD-K\ NPs-1550} \times I_{1550nm} \times \Phi_{EuB6@RGD-K\ NPs-1550\ +\ OH}$…(2)

$Area_{fluo-EuB6@RGD-K\ NPs\ -1550\ +\ APF} = 7214130$

The fluorescence area in the range of 500 to 600 nm was integrated using a photoluminescence spectrometer (Edinburgh, FLS920) at a slit width of 3 nm for both excitation and emission. The absorbance values were measured from the UV-visible-NIR spectrum. The hydroxyl radical generation quantum yield of $EuB_6$@RGD-K NPs @1550 nm is obtained by dividing the equations (1)/(2), and then by substituting the corresponding values in the above equations. The

$\Phi_{EuB6@RGD\text{-}K\ NPs\ -1550}$ for the quantum yield of hydroxyl radical formation is obtained to be **0.198** @1550 nm**.**

**Supplementary references**

[S1] M. Wainright, H. Mohr, W.H. Walker, *J. Photochem Photobiol*, **2007**, *86*, 45.
[S2] Y. Zhong, Z. Ma, S. Zhu, J. Yue, M. Zhang, A. L. Antaris, J. Yuan, R. Cui, H. Wan, Y. Zhou, W. Wang, N. F. Huang, J. Luo, Z. Hu, H. Dai, *Nature Communications* **2017**, *8*, 737.
[S3] L. Sun, J. R. Bolton, *J. Phys. Chem.* **1996,** *100,* 4127-4134.
